# Effective length correction factor of disc-buckle type scaffolding by considering joint bending stiffness and geometrical size

**DOI:** 10.1371/journal.pone.0276340

**Published:** 2022-10-20

**Authors:** Jinfeng Dong, Haiqing Liu, Ming Lei, Zimu Chen, Liang Wang, Lixin Guo, Xiangen Gong, Zhou Fang

**Affiliations:** 1 Department of School of Civil Engineering, Liaoning Technical University, Fuxin, Liaoning, China; 2 Department of Northeast Company, China Construction Fifth Engineering Division Corp., Ltd., Shenyang, Liaoning, China; Universita degli Studi di Napoli Federico II, ITALY

## Abstract

Based on the theory of sway frame column, the equation of the effective length factor was derived in this paper. Combined with the characteristics of semi-rigid joints, the linear stiffness correction factor of horizontal bar was introduced, and the equation of effective length correction factor was obtained. By using MATLAB programming method, the three-dimensional relationship between the effective length correction factor and the influencing factors was obtained, and the entire process of the stability bearing capacity of the disc-buckle type high support system was described in detail, which improves the stability calculation theory of the high support system. The influence of setting parameters, joint bending stiffness, geometrical size, and material properties on the effective length correction factor is studied. Simultaneously, the joint bending stiffness of semi-rigid joints is determined. The area of the effective length correction factor is analyzed to optimize the design of the setting scheme using horizontal bars and vertical poles of different sizes. The results show that the lift height significantly affects the effective length correction factor during the load bearing process; the factor decreases with increasing lift height. Large transverse and longitudinal distances influence this rule during the initial load bearing. When the joint bending stiffness is less than 100 (kN·m)/rad, the effective length correction factor decreases rapidly with an increase in joint bending stiffness. When the joint bending stiffness is greater than 100 (kN·m)/rad, the effective length correction factor is unaffected by the joint bending stiffness. When the joint bending stiffness is large at initiation of loading, the effective length correction factor decreases with an increase in the outer diameter of the horizontal bar. When the joint bending stiffness is small, the effective length correction factor increases with an increase in the section size of the vertical pole. Therefore, the outer diameter of the horizontal bar significantly affects the effective length correction factor, and a larger diameter is more conducive to the overall stability. Furthermore, the elastic modulus effects the effective length correction factor for the unstable support system.

## 1 Introduction

With increasing complexity of the building functional requirements, large spatial structure forms continue to emerge. However, the diversification of spatial structure forms has brought technical problems to construction. New requirements for the safety and stable construction of high support system need to be put forward. Disc-buckle type steel scaffolding has been widely used in construction. However, due to the imperfect stability calculation theory of high support systems and excessive dependence on experienced formulation of the construction scheme, safety accidents occur frequently. The method for the stability calculation of formwork support has been given in the “Technical specification for safety of disk lock steel tubular scaffold in construction” [1] and “Standard” [2], but this calculation method is questioned by scholars [3–5]. Research shows that the connection joints in the disc-buckle type formwork support system have semi-rigid connection characteristics and the moment-rotation curve of the joints is nonlinear [6–8]. Several studies have focused on the effective length correction factor of fastener-styles and other formwork supports [9–11]; the fixed value of the initial joint bending stiffness was used when considering the semi-rigid joints. However, studies on the influence of semi-rigid joints on the effective length correction factor are scarce. Zeng FK et al. [12] proposed that the effective length factor of template bracket should consider the semi-rigid joint and be calculated based on the sway frame theory; the rationality of the sway frame theory was proved by comparison with the experimental results. He XP [13] considered and analyzed the effective length factor of the vertical pole of the frame with and without lateral displacement and found that the stability of the sway frame under torsional shear was poor. Chen ZH et al. [14–16] derived the effective length factor of semi-rigid connection with lateral displacement by considering the semi-rigid connection; subsequently, they applied it to the calculation of stability bearing capacity. Lu ZR et al. [17] calculated the bearing capacity of the full fastener support frame without scissors by taking the semi-rigid nature and the constraints of horizontal bars into account and using the three-point rotation constraint single-bar stability theory. The theoretical effective length correction factor of frame columns was obtained by the sway steel frame structure [18–22]. Due to its unique structural form, the section size of a high support system was small. In the process of manufacturing, transportation, and use, the section size has geometric imperfections and the material properties differs. The setting parameters, joint bending stiffness, geometrical size and material properties have an influence on the effective length correction factor. In the early stage, the author conducted a detailed experimental study of the semi-rigid of the joints in the support system, proved the characteristics of semi-rigid joints, and conducted a parametric study on the stability of the overall structure [23, 24]. However, the stability theory, that is, the value of effective length correction factor and the influencing factors have not been studied. In this study, the analysis of the theoretical stability bearing capacity of the sway frame column was performed. Through this analysis and by considering the semi-rigid joint constraint effect of the horizontal bar [25, 26], the cloud diagram effective length correction factor was obtained. This realized the refined description of the process of the stability bearing of the high support system and improves the structural stability calculation theory. Based on the calculation equation of the effective length correction factor, the influence of relevant parameters, such as setting parameters, joint bending stiffness, geometrical size, and material properties, on the effective length correction factor was studied. This can help provide a theoretical basis and technical support for the formulation of the relevant specifications and construction schemes of the disc-buckle type high support system.

## 2 Equation derivation of effective length correction factor

### 2.1 Equation for effective length factor

**Fig 1:**
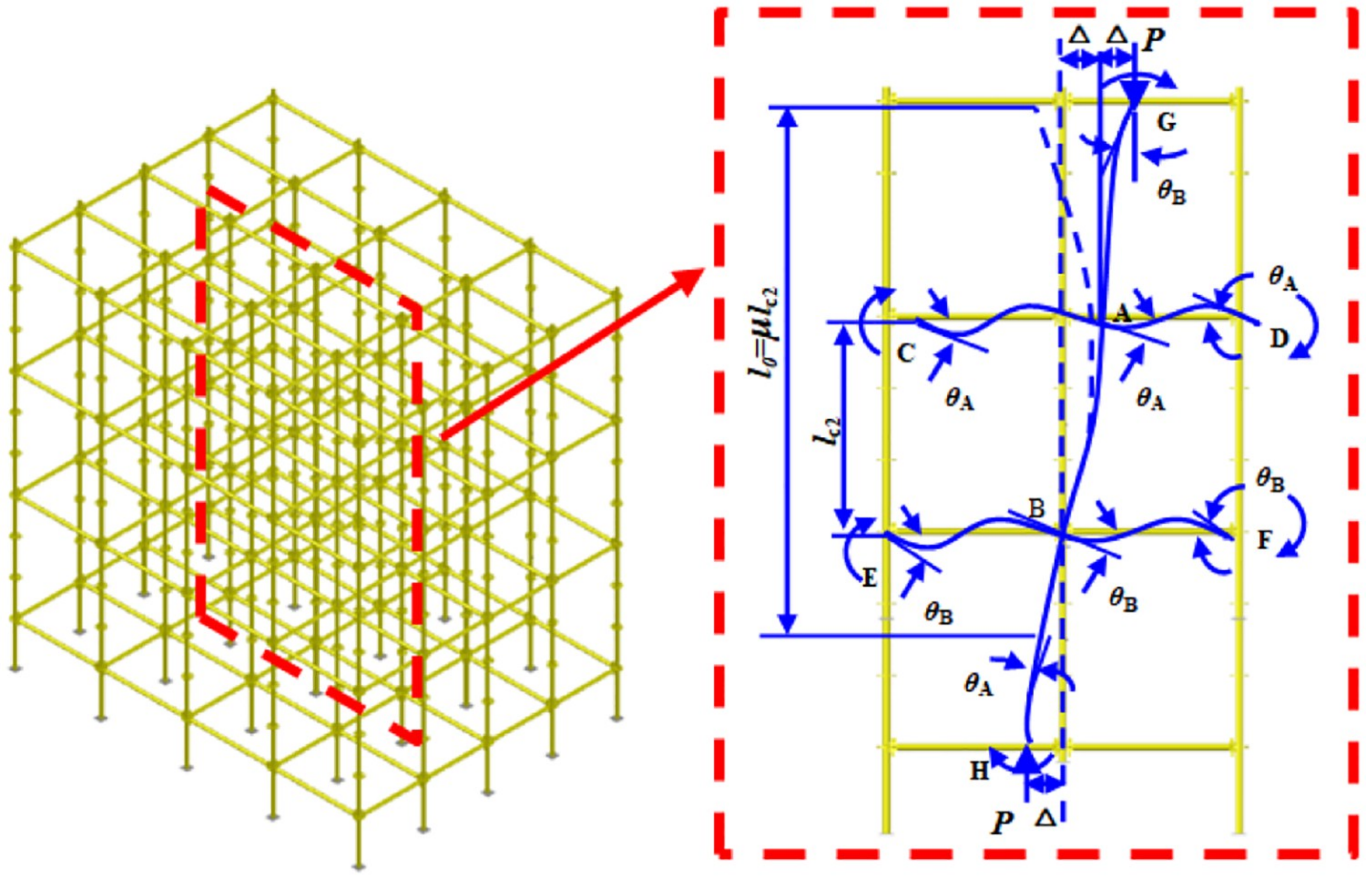
Calculation diagram of the substructure of the sway formwork support.

Fig 1 shows the multi-layer and multi-span sway high formwork support and the substructure of the sway formwork support. To determine the effective length factor of the AB vertical pole, the basic assumption of an approximate method is considered, described as follows. (1) In the template support, the AB vertical pole and the connected upper and lower two vertical poles AG and BH buckle simultaneously. (2) When the formwork support buckles, the rotation angles at both ends of each horizontal bar on the same layer are equal and in the same directions. (3) The unbalanced torque at the horizontal bar end generated at the joint during buckling is proportionally distributed to the vertical pole end according to the linear stiffness to balance it. (4) The change of the axial pressure of the vertical pole during buckling is disregarded. (5) The influence of axial force in horizontal bar is disregarded.

The moment of force of the horizontal bar and vertical pole ends related to joint A are established as *M*_AB_ = (*EI*_c2_/*l*_c2_)[*Cθ*_A_ + *Sθ*_B_ − (*C* + *S*)*ρ*_2_] and *M*_AG_ = (*EI*_c1_/*l*_c1_)[*Cθ*_A_ + *Sθ*_B_ − (*C* + *S*)*ρ*_1_], where $\rho_{1}=\frac{\Delta }{l_{\text{c}1}}$, $\rho_{2}=\frac{\Delta }{l_{\text{c}2}}$, *ρ*_1_ = *ρ*_2_, and $M_{\text{AG}}=M_{\text{AB}}\frac{I_{\text{c}1}/l_{\text{c}1}}{I_{\text{c}2}/l_{\text{c}2}}$, *M*_AC_ = 6(*EI*_b1_/*l*_b1_)*θ*_A_, *M*_AD_ = 6(*EI*_b2_/*l*_b2_)*θ*_A_.

The equilibrium equation of joint A is established as ∑*M*_A_ = 0, that is, *M*_AB_ + *M*_AG_ + *M*_AC_ + *M*_AD_ = 0 and Eq (1) was obtained.


$$C\theta_{\text{A}}+S\theta_{\text{B}}-\left(C+S\right)\rho_{2}+\frac{6\left(I_{\text{b}1}/l_{\text{b}1}+I_{\text{b}2}/l_{\text{b}2}\right)\theta_{\text{A}}}{I_{\text{c}1}/l_{\text{c}1}+I_{\text{c}2}/l_{\text{c}2}}=0$$ (1)

The ratio *K*_1_ was introduced. It is defined as the sum of linear stiffness of the horizontal bar connected to the upper end of the AB vertical pole to the linear stiffness of the vertical pole. Thus, Eq (1) can be written as *Cθ*_A_ + *Sθ*_B_ − (*C* + *S*)*ρ*_2_ + 6*K*_1_*θ*_A_ = 0. Reorganizing the results, we obtain Eq (2):

$$\left(C+6K_{1}\right)\theta_{\text{A}}+S\theta_{\text{B}}-\left(C+S\right)\rho_{2}=0.$$ (2)

Similarly, the moment equilibrium equation of joint B can be established and Eq (4) can be obtained by introducing the linear stiffness ratio *K*_2_ related to the lower end constraint of AB vertical pole.

First, torques of horizontal bars and vertical poles end related to joint B are established as *M*_BA_ = (*EI*_c2_/*l*_c2_)[*Cθ*_B_ + *Sθ*_A_ − (*C* + *S*)*ρ*_2_] and *M*_BH_ = (*EI*_c3_/*l*_c3_)[*Cθ*_B_ + *Sθ*_A_ − (*C* + *S*)*ρ*_3_], where $\rho_{2}=\frac{\Delta }{l_{\text{c}2}}$, $\rho_{3}=\frac{\Delta }{l_{\text{c}3}}$, *ρ*_2_ = *ρ*_3_, and $M_{\text{BH}}=M_{\text{BA}}\frac{I_{\text{c}3}/l_{\text{c}3}}{I_{\text{c}2}/l_{\text{c}2}}$, *M*_BE_ = 6(*EI*_b3_/*l*_b3_)*θ*_B_ and *M*_BF_ = 6(*EI*_b4_/*l*_b4_)*θ*_B_. Subsequently, the equilibrium equation of joint B is established as ∑*M*_B_ = 0, that is, *M*_BH_ + *M*_BA_ + *M*_BE_ + *M*_BF_ = 0, and Eq (3) was obtained.


$$C\theta_{\text{B}}+S\theta_{\text{A}}-\left(C+S\right)\rho_{2}+\frac{6\left(I_{\text{b}3}/l_{\text{b}3}+I_{\text{b}4}/l_{\text{b}4}\right)\theta_{\text{B}}}{I_{\text{c}2}/l_{\text{c}2}+I_{\text{c}3}/l_{\text{c}3}}=0$$ (3)

The ratio *K*_2_ of the sum of the linear stiffness of the horizontal bar connected to the lower end of the AB vertical pole to the linear stiffness of the vertical pole was introduced, and Eq (3) can be written as *Cθ*_B_ + *Sθ*_A_ − (*C* + *S*)*ρ*_2_ + 6*K*_2_*θ*_B_ = 0. Reorganizing the results, we obtain Eq (4):

$$S\theta_{\text{A}}+\left(C+6K_{2}\right)\theta_{\text{B}}-\left(C+S\right)\rho_{2}=0$$ (4)

To establish the equilibrium equation of the vertical pole itself, *M*_AB_ + *M*_BA_ + *P*Δ = 0, where Δ = *ρ*_2_*l*_c2_ and *P* = *k*^2^*EI*_c2_. Furthermore, *P*Δ = *k*^2^*EI*_c2_*ρ*_2_*l*_c2_ = (*EI*_c2_/*l*_c2_)(*kl*_c2_)^2^*ρ*_2_ and (*EI*_c2_/*l*_c2_)[*Cθ*_A_ + *Sθ*_B_ − (*C* + *S*)*ρ*_2_] + (*EI*_c2_/*l*_c2_)[*Cθ*_B_ + *Sθ*_A_ − (*C* + *S*)*ρ*_2_] + (*EI*_c2_/*l*_c2_)(*kl*_c2_)^2^ *ρ*_2_ = 0.

Reorganizing results in Eq (5):

$$\left(C+S\right)\left(\theta_{\text{A}}+\theta_{\text{B}}\right)-\left[2\left(C+S\right)-{\left(kl_{\text{c}2}\right)}^{2}\right]\rho_{2}=0$$ (5)

Adding Eqs (2) and (4), we obtain Eq (6):

$$\left(C+S\right)\left(\theta_{\text{A}}+\theta_{\text{B}}\right)-2\left(C+S\right)\rho_{2}=-6\left(K_{1}\theta_{\text{A}}+K_{2}\theta_{\text{B}}\right)$$ (6)

Subsequently, Eq (7) is obtained as:

$$-6K_{1}\theta_{\text{A}}-6K_{2}\theta_{\text{B}}+{\left(kl_{\text{c}2}\right)}^{2}\rho_{2}=0$$ (7)

From the linear Eqs (2), (4) and (7), the buckling Eq (8) of the vertical pole of formwork support with lateral displacement can be obtained as:

$$\left|\begin{array}{ccc} \left(C+6K_{1}\right) & S & -\left(C+S\right)\\ S & \left(C+6K_{2}\right) & -\left(C+S\right)\\ -6K_{1} & -6K_{2} & {\left(kl_{\text{c}2}\right)}^{2} \end{array}\right|=0.$$ (8)

The trigonometric functions of *C* and *S* are substituted and expressed by *μ* = *π*/*kl*_c2_, after which the buckling Eq (9) is obtained as:

$$\left[36K_{1}K_{2}-{\left(\pi /\mu \right)}^{2}\right]\text{tan}\left(\pi /\mu \right)+6\left(K_{1}+K_{2}\right)\pi /\mu =0.$$ (9)

GB 50017–2017 Appendix E.0.2 gives the effective length factor *μ* of laterally shifted multi-story and multi-span rigid frame columns, which depends on the constraint parameters *K*_1_ and *K*_2_ Additionally, the value of *μ* can be determined by the following practical calculation Eq (10)

$$\mu =\sqrt{\frac{7.5K_{1}K_{2}+4\left(K_{1}+K_{2}\right)+1.52}{7.5K_{1}K_{2}+K_{1}+K_{2}}}.$$ (10)

### 2.2 Equation for effective length correction factor

Let the joint bending stiffness be *R*, which is obtained by the semi-rigid stiffness curve, and the rotation angle of the double curvature bar end is *θ*_A_ = *θ*_D_ and *M*_AD_ = *M*_DA_. By substituting these into Eqs (11) and (12) is obtained.

$$M_{\text{AD}}=\frac{EI_{\text{b}}}{l_{\text{b}}}\left[4\left(\theta_{\text{A}}-\frac{M_{\text{AD}}}{R}\right)+2\left(\theta_{\text{D}}-\frac{M_{\text{DA}}}{R}\right)\right],$$ (11)

$$M_{\text{AD}}=\frac{EI_{\text{b}}}{l_{\text{b}}}\left[4\theta_{\text{A}}-4\frac{M_{\text{AD}}}{R}+2\theta_{\text{D}}-2\frac{M_{\text{DA}}}{R}\right],$$

$$M_{\text{AD}}=\frac{EI_{\text{b}}}{l_{\text{b}}}\left[6\theta_{\text{A}}-6\frac{M_{\text{AD}}}{R}\right],$$

$$M_{\text{AD}}=\frac{6EI_{\text{b}}}{l_{\text{b}}}\left[\theta_{\text{A}}-\frac{M_{\text{AD}}}{R}\right],$$

$$M_{\text{AD}}=\frac{6EI_{\text{b}}}{l_{\text{b}}}\cdot \frac{1}{1+6EI_{\text{b}}/l_{\text{b}}R}\cdot \theta_{\text{A}},$$ (12)

where *l*_b_ denotes the transverse and longitudinal distance; *R* denotes joint bending stiffness; *I*_b_ denotes the cross sectional moment of inertia of horizontal bar; and *E* denotes the elastic modulus of horizontal bar.

The linear stiffness correction factor *α* of horizontal bar can be obtained from Eq (12), as shown in Eq (13).


$$\alpha =\frac{1}{1+6EI_{\text{b}}/l_{\text{b}}R}$$ (13)

Then, the effective length correction factor *μ*_mod_ is obtained, as shown in Eq (14).

$$\mu_{\text{mod}}=\sqrt{\frac{7.5\alpha K_{1}\alpha K_{2}+4(\alpha K_{1}+\alpha K_{2})+1.52}{7.5\alpha K_{1}\alpha K_{2}+\alpha K_{1}+\alpha K_{2}}},$$ (14)

where, *μ*_mod_ denotes the effective length correction factor; *K*_1_ denotes the ratio of the sum of horizontal bar linear stiffness of the upper end of vertical pole to the vertical pole linear stiffness, $K_{1}=\frac{I_{\text{b}1}/l_{\text{b}1}+I_{\text{b}2}/l_{\text{b}2}}{I_{\text{c}1}/l_{\text{c}1}+I_{\text{c}2}/l_{\text{c}2}}$; *K*_2_ denotes the ratio of the sum of horizontal bar linear stiffness of the lower end of vertical pole to the vertical pole linear stiffness, $K_{2}=\frac{I_{\text{b}3}/l_{\text{b}3}+I_{\text{b}4}/l_{\text{b}4}}{I_{\text{c}2}/l_{\text{c}2}+I_{\text{c}3}/l_{\text{c}3}}$; and *α* denotes the linear stiffness correction factor of horizontal bar, $\alpha =\frac{1}{1+6EI_{\text{b}}/l_{\text{b}}R}$.

According to Appendix A of the Technical specification for safety of disk lock steel tubular scaffold in construction JGJ231-2010 [1], the specifications and section property of horizontal bars and vertical poles of disc-buckle type scaffolds are summarized in Table 1.

**Table 1:** Specification and section property of horizontal bars and vertical poles.

Type	Model	Length/m	Specification	Inertial Moment *I*(m^4^)
**Horizontal bar**	A-SG	0.3,0.6,0.9,1.2,1.5,1.8,2.0	*ϕ*48 × 2.5	9.28 × 10^−8^
	B-SG	0.3,0.6,0.9,1.2,1.5,1.8,2.0	*ϕ*42 × 2.5	6.07 × 10^−8^
**Vertical pole**	A-LG	0.5,1.0,1.5,2.0,2.5,3.0	*ϕ*60 × 3.2	23.10 × 10^−8^
	B-LG	0.5,1.0,1.5,2.0,2.5,3.0	*ϕ*48 × 3.2	11.36 × 10^−8^

In high support system, the lift height and transverse and longitudinal distance are generally set in the same setting scheme; furthermore, the specifications of the horizontal bar and vertical pole are fixed. Therefore, in general, *K*_1_ = *K*_2_ = *k*, *l*_b1_ = *l*_b2_ = *l*_b3_ = *l*_b4_ = *l*_b_, *l*_c1_ = *l*_c2_ = *l*_c3_ = *l*_c4_ = *l*_c_, *I*_b1_ = *I*_b2_ = *I*_b3_ = *I*_b4_ = *I*_b_, *I*_c1_ = *I*_c2_ = *I*_c3_ = *I*_c4_ = *I*_c_.

Hence, Eq (14) can be written as, $\mu_{\text{mod}}=\sqrt{\frac{7.5\alpha k\alpha k+4(\alpha k+\alpha k)+1.52}{7.5\alpha k\alpha k+\alpha k+\alpha k}}$, where *K* = *αk* and $\alpha =\frac{1}{1+6EI_{\text{b}}/l_{\text{b}}R}=\frac{l_{\text{b}}R}{l_{\text{b}}R+6EI_{\text{b}}}$.

Eq (15) is obtained:

$$K=\alpha k=\frac{I_{\text{b}}}{I_{\text{c}}}\times \frac{l_{\text{c}}R}{l_{\text{b}}R+6EI_{\text{b}}}.$$ (15)

From Eq (15), it can be inferred that when the type of horizontal bar and vertical pole is determined, only the joint bending stiffness, longitudinal and transverse distance, and lift height are variables; essentially, the effective length correction factor *μ*_mod_ is related to three variables.

### 2.3 Condition analysis of *K* value

There are four primary conditions for the value of *K*, described as follows.

Condition 1: the horizontal bar adopts the model of A-SG, *E* = 2.06 × 10^8^kN/m^2^ and *I*_bA_ = 9.28 × 10^−8^m^4^; the vertical pole adopts the model of A-LG, *E* = 2.06 × 10^8^kN/m^2^ and *I*_cA_ = 23.10 × 10^−8^m^4^. Therefore, $K_{\text{I}}=\alpha k=\frac{I_{\text{bA}}}{I_{\text{cA}}}\times \frac{l_{\text{c}}R}{l_{\text{b}}R+6EI_{\text{bA}}}=\frac{9.28}{23.10}\times \frac{l_{\text{c}}R}{l_{\text{b}}R+114.7008}$.

Condition 2: the horizontal bar adopts the model of A-SG, *E* = 2.06 × 10^8^kN/m^2^ and *I*_bA_ = 9.28 × 10^−8^m^4^; the vertical pole adopts the model of B-LG, *E* = 2.06 × 10^8^kN/m^2^ and *I*_cB_ = 11.36 × 10^−8^m^4^. Therefore, $K_{\text{II}}=\alpha k=\frac{I_{\text{bA}}}{I_{\text{cB}}}\times \frac{l_{\text{c}}R}{l_{\text{b}}R+6EI_{\text{bA}}}=\frac{9.28}{11.36}\times \frac{l_{\text{c}}R}{l_{\text{b}}R+114.7008}$.

Condition 3: the horizontal bar adopts the model of B-SG, *E* = 2.06 × 10^8^kN/m^2^ and *I*_bB_ = 6.07 × 10^−8^m^4^; the vertical pole adopts the model of A-LG, *E* = 2.06 × 10^8^kN/m^2^ and *I*_cA_ = 23.10 × 10^−8^m^4^. Therefore, $K_{\text{III}}=\alpha k=\frac{I_{\text{bB}}}{I_{\text{cA}}}\times \frac{l_{\text{c}}R}{l_{\text{b}}R+6EI_{\text{bB}}}=\frac{6.07}{23.10}\times \frac{l_{\text{c}}R}{l_{\text{b}}R+75.0252}$.

Finally, condition 4: the horizontal bar adopts the model of B-SG, *E* = 2.06 × 10^8^kN/m^2^ and *I*_bB_ = 6.07 × 10^−8^m^4^; the vertical pole adopts model of B-LG, *E* = 2.06 × 10^8^kN/m^2^ and *I*_cB_ = 11.36 × 10^−8^m^4^. Therefore, $K_{\text{IV}}=\alpha k=\frac{I_{\text{bB}}}{I_{\text{cB}}}\times \frac{l_{\text{c}}R}{l_{\text{b}}R+6EI_{\text{bB}}}=\frac{6.07}{11.36}\times \frac{l_{\text{c}}R}{l_{\text{b}}R+75.0252}$.

## 3 Joint bending stiffness

### 3.1 Experimental analysis on joint bending stiffness

#### 3.1.1 Geometrical size of socket connections

**Fig 2:**
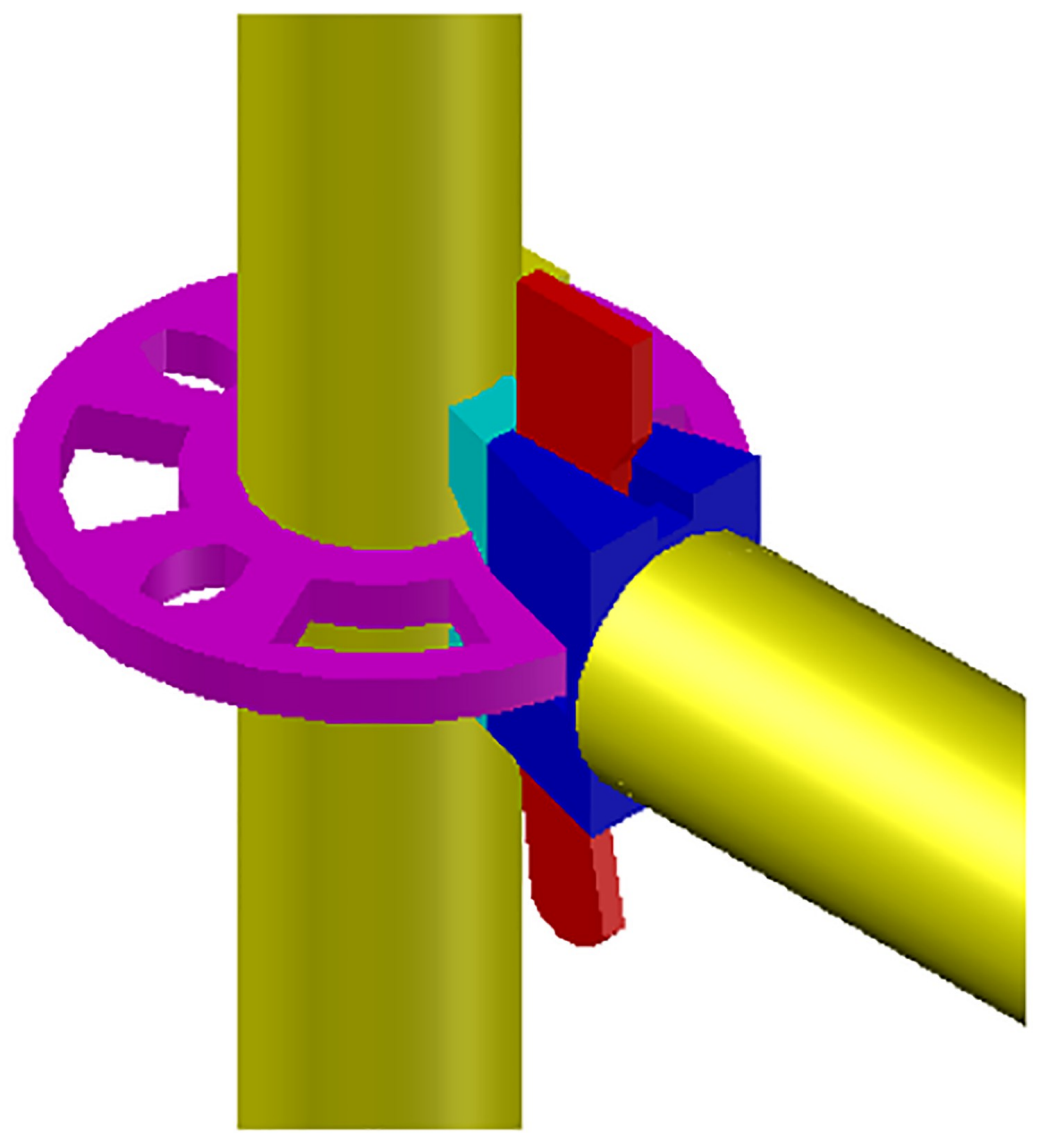
Disc-buckled type connection joint.

The connection joints of the disc-buckle type formwork support comprised the disk-plate welded to the vertical pole, wedge head welded to the horizontal bar, and the wedge inserted into the wedge head welded to the end of the diagonal bracing, as shown in Fig 2. The material of the horizontal bar was Q235A, that of the vertical pole was Q345A [27], that of the wedge head was ZG230-450, and those of the disk-plate and wedge were ZG230-450 and Q235B, respectively. The spacing of the disk-plate of the vertical pole was 0.5 m, and the length of the horizontal bar was 0.3 m.

**Fig 3:**
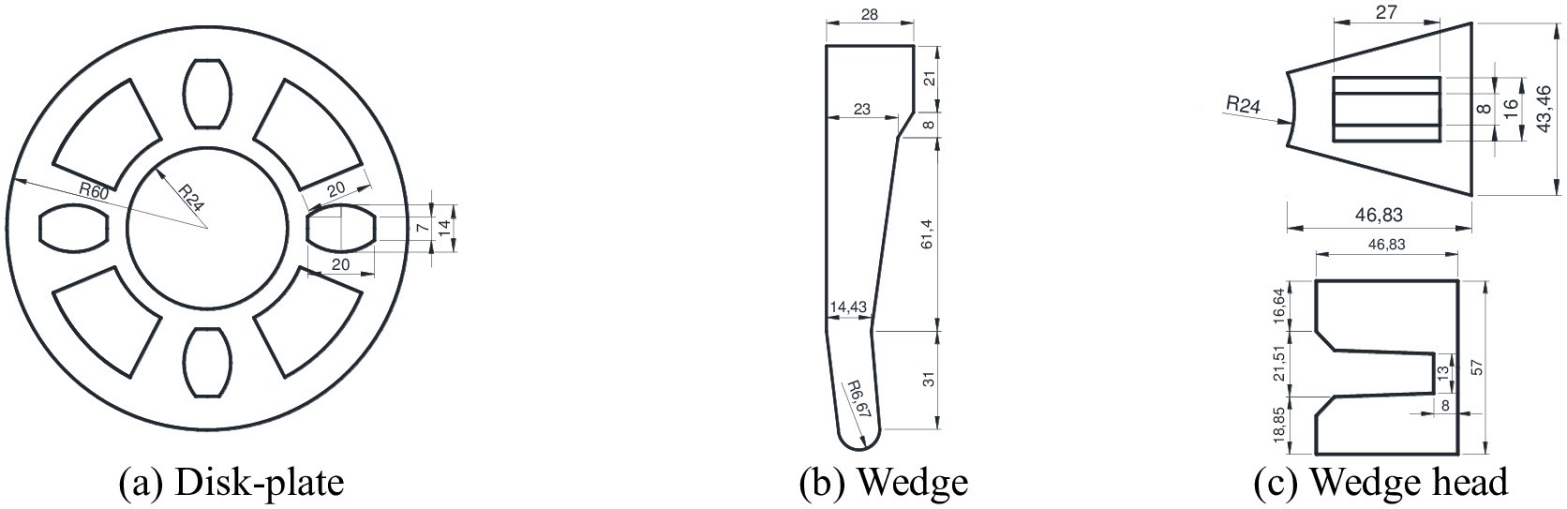
Geometrical size of the connection joints (unit: mm). (a) Disk-plate. (b) Wedge. (c) Wedge head.

The geometrical size of the connection joints used at the construction site is shown in Fig 3. The outer diameter and wall thickness of the horizontal bar were 48 and 2.5 mm, respectively, and the outer diameter and wall thickness of the vertical pole were 48 and 3.2 mm, respectively. According to the specification [1], the allowable deviation of the wall thickness of steel pipe should be ± 0.1 mm. The size of the disk-plate is shown in Fig 3(a). The outer diameter of the disk-plate was 60 mm and the length of the bolt hole was 20 mm. The size of the wedge is shown in Fig 3(b); there are different thicknesses of the wedges. The specific thickness of the wedge was measured and found to be 5.5–6.5 mm. As shown in Fig 3(c), the bolt hole size of the upper and lower wedge heads were 8 mm × 27 mm and 16 mm × 27 mm, respectively.

**Fig 4:**
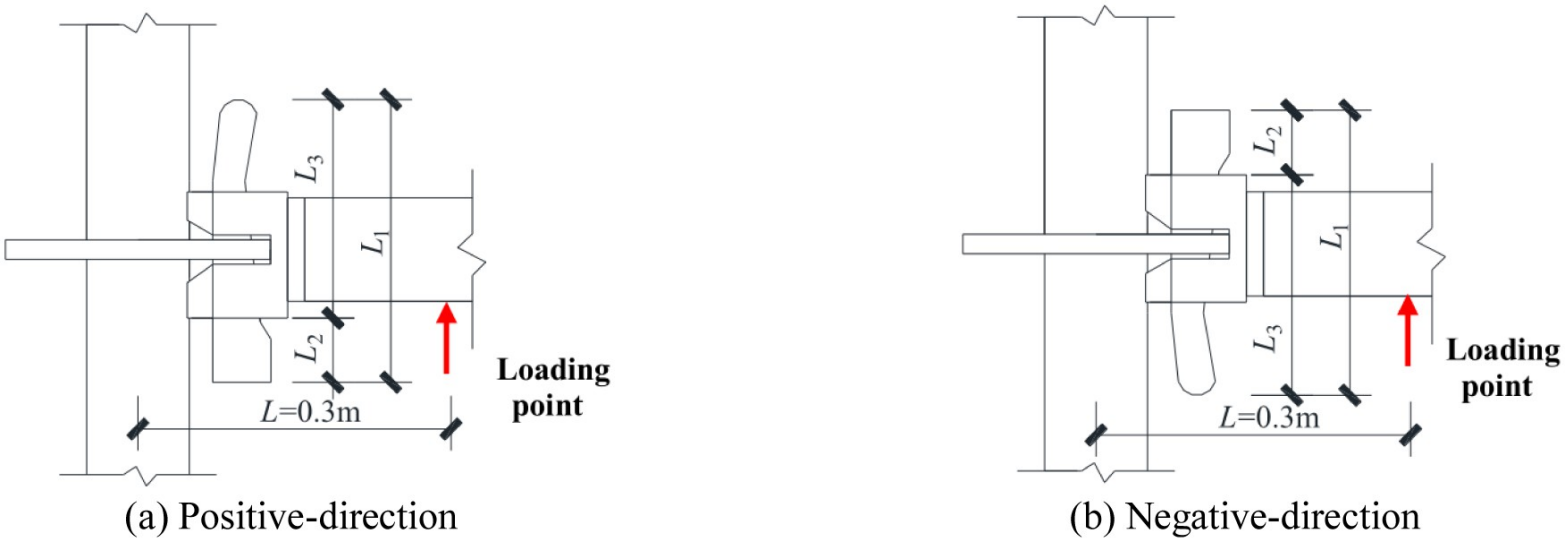
Dimensions schematic of the wedge insertion state. (a) Positive-direction. (b) Negative-direction.

All components were obtained from the construction site. Due to the nonstandard production and recycling, the dimensions of components differed, which resulted in different configurations of each experimental joint. The specific differences are shown in the depth *L*_3_ of the wedge insertion in the disk-plate, the thickness of the wedge *b* and the disk-plate *t*_P_. The wedge insertion state of the positive- and negative-directions are shown in Fig 4. Detailed dimensions of the insertion depth, thickness of the wedge and the disk-plate are listed in Table 2.

**Table 2:** Detailed dimensions of the insertion depth, thickness of the wedge and the disk-plate.

No.	*L*_2_/mm	*L*_3_/mm	*L*_1_/mm	*b*/mm	*t*_P_/mm	No.	*L*_2_/mm	*L*_3_/mm	*L*_1_/mm	*b*/mm	*t*_P_/mm
**P-SJ1**	30	98	128	5.80	10.02	**N-SJ1**	39	89	128	5.79	9.72
**P-SJ2**	37	91	128	6.09	9.66	**N-SJ2**	38	90	128	5.61	9.71
**P-SJ3**	39	89	128	5.60	9.80	**N-SJ3**	40	88	128	5.77	9.81
**P-SJ4**	34	94	128	6.00	9.65	**N-SJ4**	37	91	128	5.81	9.74
**P-SJ5**	39	89	128	6.00	9.79	**N-SJ5**	35	93	128	5.63	9.77
**P-SJ6**	30	98	128	5.67	9.72	**N-SJ6**	41	87	128	6.10	9.80
**P-SJ7**	28	100	128	5.95	10.00	**N-SJ7**	41	87	128	6.19	9.85
**P-SJ8**	33	95	128	5.63	9.55	**N-SJ8**	35	93	128	6.08	10.11

The joint bending state consists of positive- and negative-direction bendings, represented by “P” and “N,” respectively, in the study. Eight groups of parallel experiments were carried out to study the effects of the positive- and negative-direction bending.

#### 3.1.2 Experimental device and loading scheme

**Fig 5:**
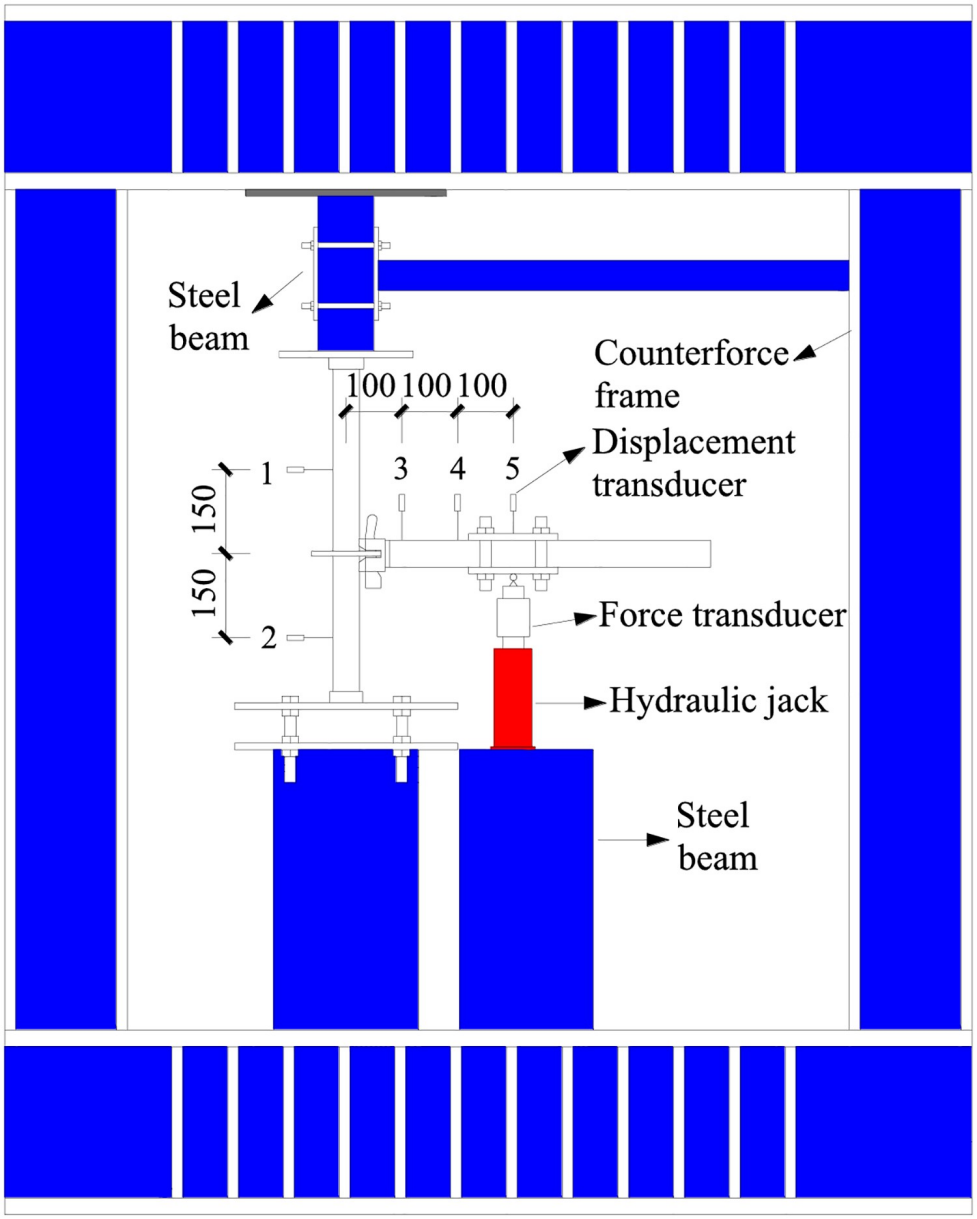
Experimental device diagram (unit: mm).

The experimental device is shown in Fig 5. The vertical bending of the connection joints of the disc-buckle type formwork support was carried out using the counterforce frame and loading mold. The upper and lower ends of the joints were fixed to the counterforce frame by the loading mold to avoid the relative slip of the connection joints during the loading process. The external force required for the loading of the specimen was provided by the 5 T hydraulic jack.

The loading mechanism was controlled by force and the process included preloading, formal loading, and unloading. Each specimen was preloaded to approximately 20% of the yield load to verify whether the experimental device and measurement equipment were operating normally. Each loading stage was 1/10-1/15 of the estimated ultimate load and the load remained in each step for 2 min. When the horizontal bar had a large angle relative to the vertical pole and the load was no longer increased, the displacement increased gradually. This was considered as the specimen of joints reaching the ultimate failure state. The formal experimental loading mechanism is listed in Table 3.

**Table 3:** Experimental loading mechanism.

**Loading force/kN**	0.1	…	0.6	0.7	0.8	…	3.4	3.6	…
**Load remaining time/min**	2	…	2	2	2	…	2	2	…

#### 3.1.3 Arrangement of measuring points

**Fig 6:**
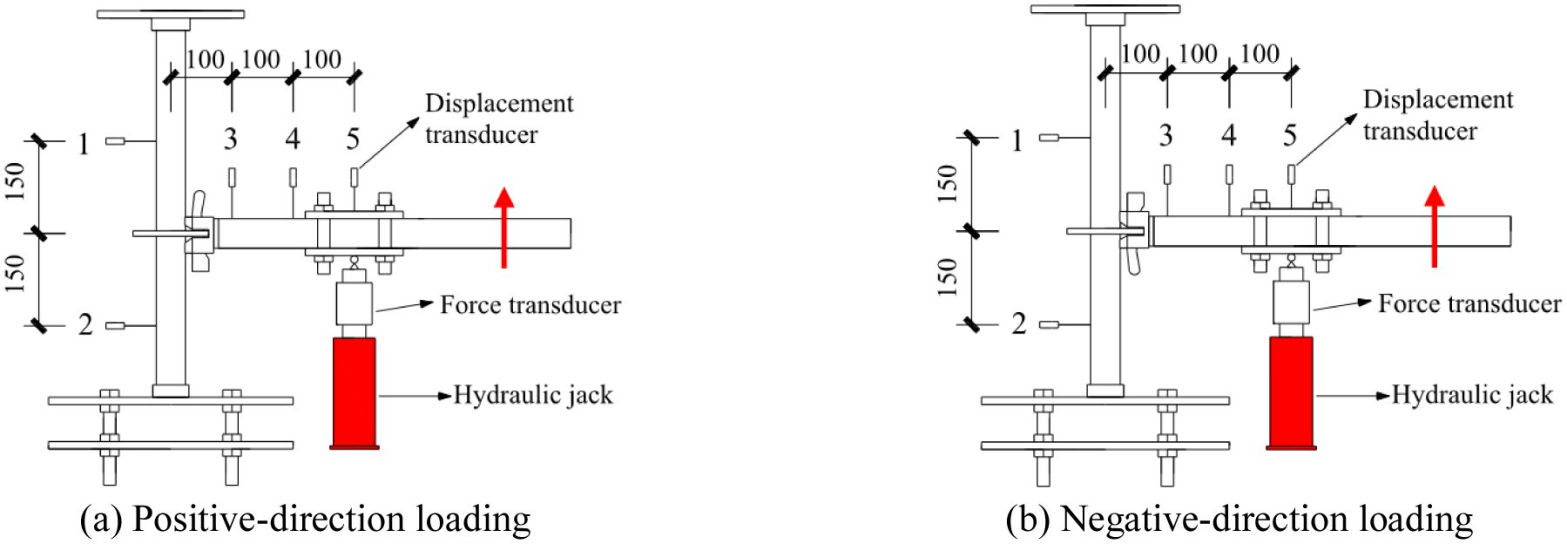
Arrangement of the displacement measuring points (unit: mm). (a) Positive-direction loading. (b) Negative-direction loading.

Five displacement transducers with measurement accuracy of 0.01 mm were used to measure the displacement of the horizontal bar and vertical pole during loading. The upper and lower measuring points of the vertical pole were set to 150 mm from the joint center, which were recorded as measuring point 1 and 2, respectively. The displacement measuring points of the horizontal bar were set 100 mm and 200 mm away from the joint center, which were recorded as measuring point 3 and 4, respectively. The loading point of the horizontal bar was set to 300 mm away from the joint center, which was recorded as measuring point 5; this measuring point was set for the calibration data. The arrangement of the measuring points is shown in Fig 6.

#### 3.1.4 Measurement method of joint rotational stiffness

Qian XJ [28] employed the bending moment–rotation angle curve to measure the parameters of disc-buckle type and bowl buckle-type formwork support joints. In this approach, the joint bending moment is taken as the product of the vertically concentrated load at loading point of the horizontal bar and the distance from the loading point to joint center, which can be calculated using Eq (16). Here, *F* denotes the vertically concentrated load at the loading point of the horizontal bar and *L* denotes the distance from the loading point to the joint center (which can be taken as 300 mm). The relative rotation angle of the joint can be obtained using Eq (17). Here, Δ_1_, Δ_2_, Δ_3_, and Δ_4_ denotes the displacement transducer reading data at measuring points 1, 2, 3, and 4, respectively. Furthermore, *d*_3–4_ denotes the straight-line distance between the measuring points 3 and 4, *d*_1_ and *d*_2_ denotes the distances from measuring points 1 and 2 to the joint center, respectively, and *θ*_1_ denotes the rotation angle of the horizontal bar section relative to the joint. A section of the vertical pole produces a rotation angle relative to the joint, which is denoted by *θ*_2_ and expressed in Eq (17). After the influence of the deformation of the vertical pole was compensated for, the angle of the joint obtained was more accurate because the vertical pole produced an elastic deformation during the loading process.


M=F×L (16)

$$\theta =\theta_{1}-\theta_{2}={\text{tan}}^{-1}\frac{\Delta_{4}-\Delta_{3}}{d_{3-4}}-{\text{tan}}^{-1}\frac{\Delta_{2}-\Delta_{1}}{d_{1}+d_{2}}$$ (17)

The bending moment–rotation angle curve of the joint was obtained by processing the data obtained from the experiment through Eqs (16) and (17).

### 3.2 Experimental results and analysis

#### 3.2.1 Failure mode

**Fig 7:**
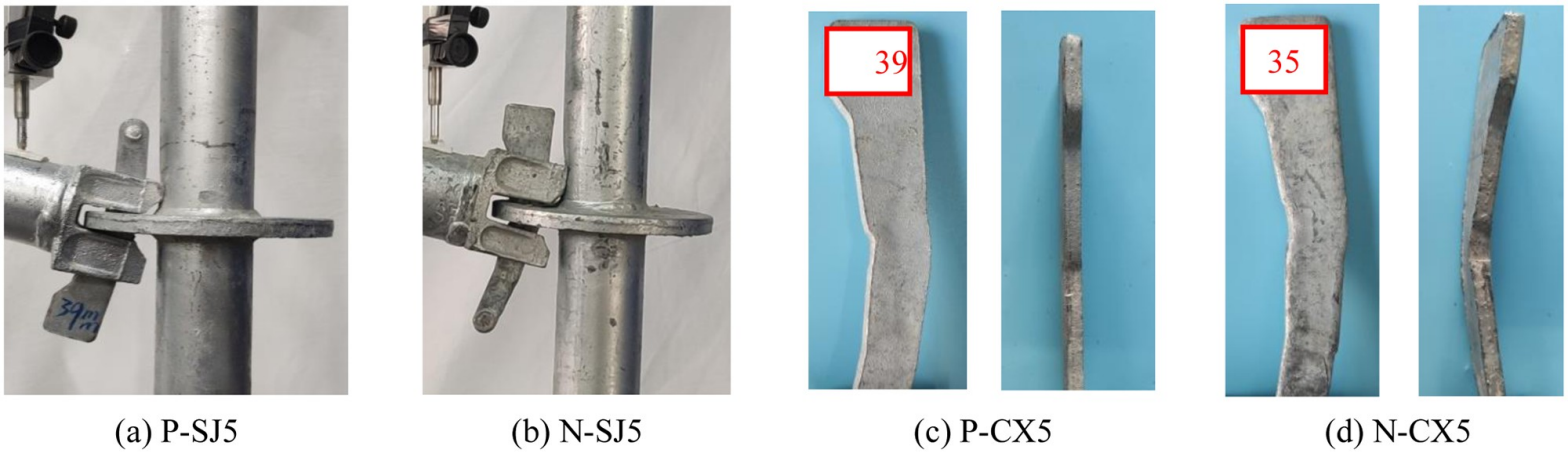
Wedge failure modes. (a) P-SJ5. (b) N-SJ5. (c) P-CX5. (d) N-CX5.

At the initial stage of loading, the specimen had no obvious deformation; the joint bending stiffness is larger when the bending moment is small (Fig 7). With increase in the joint bending moment, the joint bending stiffness decreased gradually. When the bending moment reaches a peak value, the displacement increases sharply when the bending moment increases little. At this time, the buckling failure of the joint occurs, and there is no fracture failure of the wedge and its components. Meantime, it can be observed that the contact part between the upper part of the wedge and the vertical pole has been separated, and the horizontal bar has a large rotation angle relative to the vertical pole, which can be approximately considered that the joint has entered the bending limit state. When the depth of the wedge insertion the disk-plate is relatively large, the buckling deformation of the wedge failure mode is more serious. It can be seen that the buckling deformation of the negative wedge failure mode is more serious, indicated that the performance requirements of the wedge material are higher when the joint is in the negative bending state.

#### 3.2.2 Analysis of bending capacity

**Fig 8:**
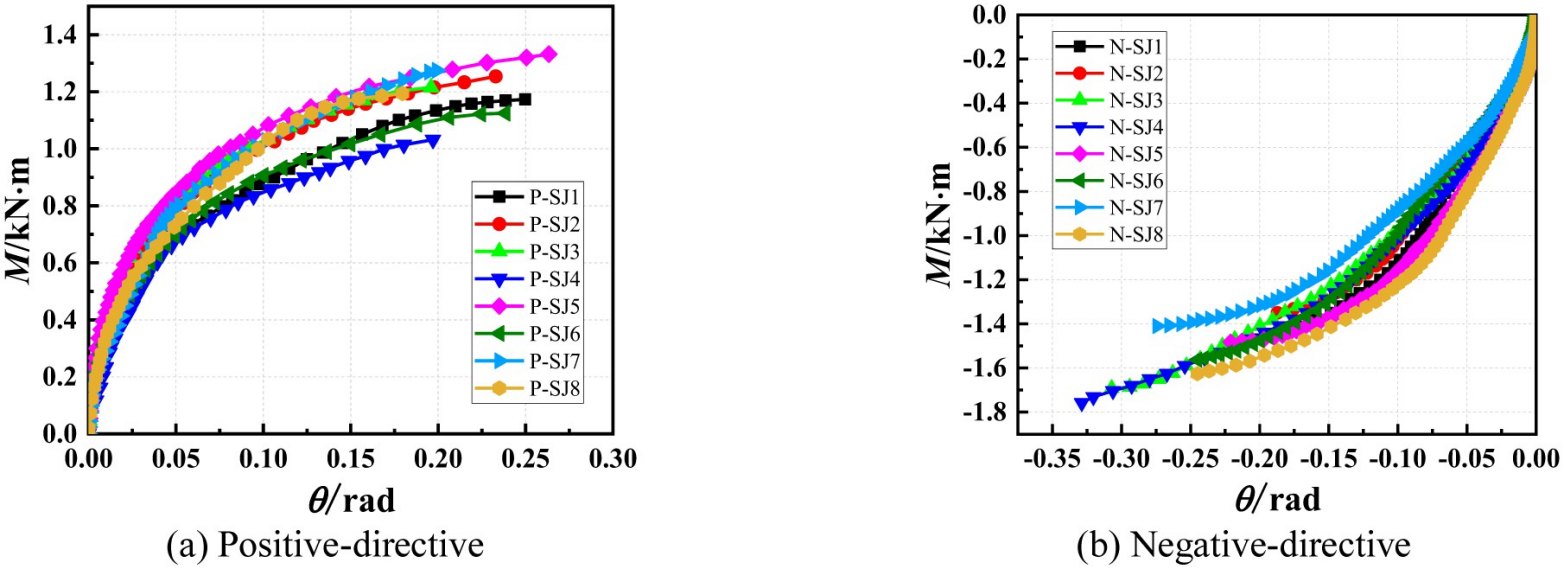
Moment–rotation curves obtained by experiments. (a) Positive-directive. (b) Negative-directive.

The moment–rotation curves corresponding to each experiment are shown in Fig 8. The bending capacity was approximately 1.2 and 1.5 kN·m in the positive and negative directions, respectively. The bending capacity in both directions were similar, and the bending capacity in the negative direction was slightly larger than that in the positive direction.

As can be seen from Fig 8(a) that the experimental data of P-SJ4 have large deviations and are not analyzed. The thickness of the wedge of P-SJ5 and P-SJ7 is almost the same, and their bending capacity is almost the same. The bending capacity of P-SJ7 with larger thickness of disk-plate is not greater than that of P-SJ5, indicated that the material constitutive of P-SJ5 wedge may be greater than that of P-SJ7, because the depth of the wedge insertion the disk-plate has no influence on the bending capacity of the positive bending joint. The bending capacity of P-SJ3, P-SJ8 and P-SJ2 is almost the same, and the thickness of the wedge of P-SJ2 is not larger than that of P-SJ3 and P-SJ8, which may be due to the small material constitutive. The bending capacity of P-SJ1 is larger than that of P-SJ6, and the depth and thickness of wedge insertion are the same. The thickness of disk-plate of P-SJ1 is much larger than that of P-SJ6. Compared with P-SJ3 and P-SJ5, the insertion depth is basically the same as the thickness of disk-plate, and the bending capacity of P-SJ5 with larger thickness of the wedge is greater than that of P-SJ3. It is shown that the positive bending capacity of joint is mainly related to the thickness of the wedge, the material constitutive of the wedge and the thickness of disk-plate, and the thickness of disk-plate is the most important factor.

As can be seen from Fig 8(b) that the experimental data of N-SJ7 have large deviations and are not analyzed. In the comparison groups of N-SJ1 and N-SJ4, N-SJ2 and N-SJ5, the thickness of the wedge and the thickness of the disk-plate are generally consistent, and the bending capacity of N-SJ4 and N-SJ5 with larger insertion depth is larger. The thickness of the wedge of N-SJ6 and N-SJ8 in the comparison groups is basically the same, and the bending capacity of N-SJ8 with larger insertion depth and thickness of the disk-plate is larger. The insertion depth of N-SJ5 and N-SJ8 was the same in the comparison group, and the bending capacity of N-SJ8 with large thickness of the wedge and disk-plate was large. It indicated that the negative bending capacity of the joint was mainly related to the depth of the wedge insertion the disk-plate, the thickness of the wedge and disk-plate.

#### 3.2.3 Characteristics of semi-rigid Joints

**Fig 9:**
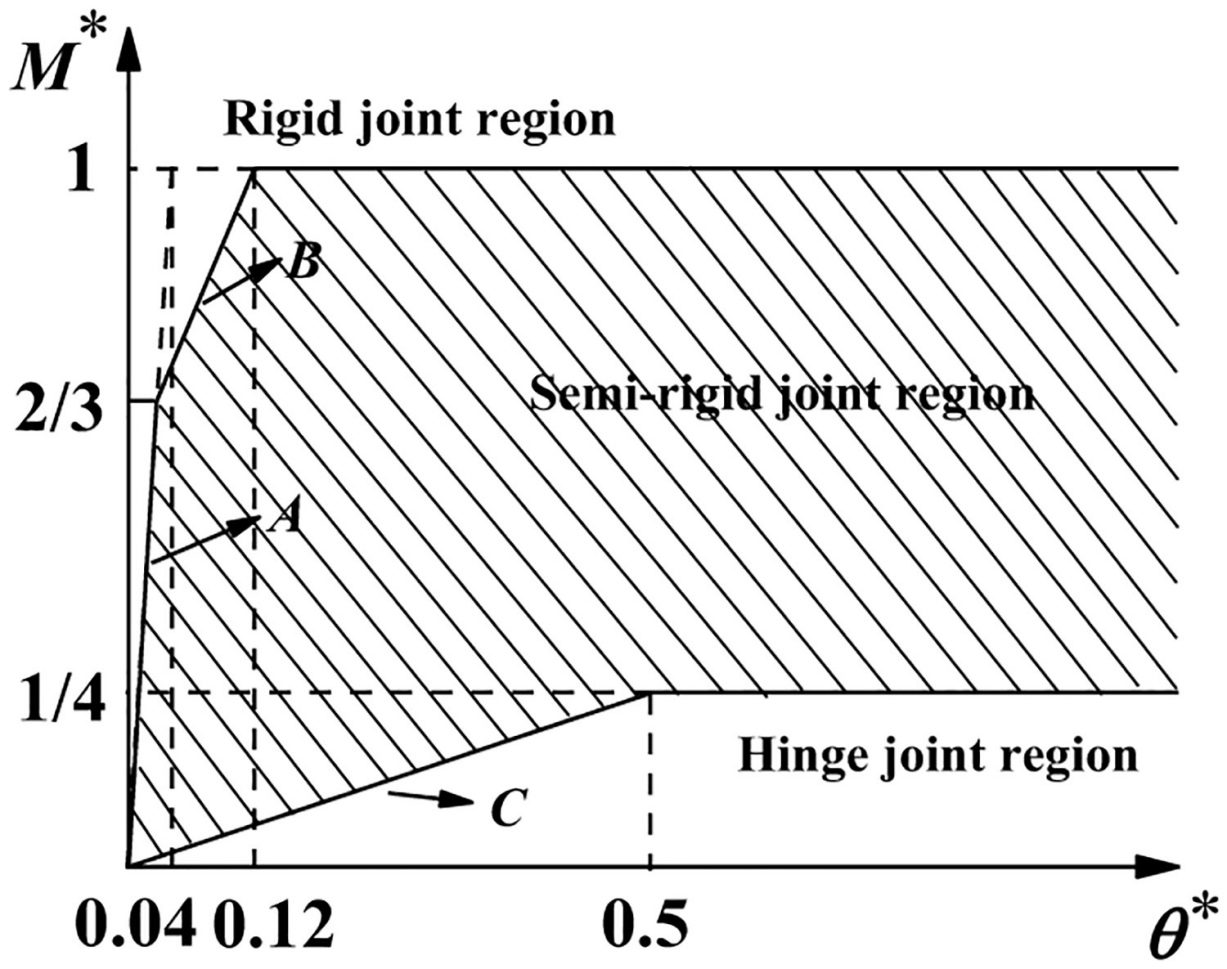
Semi-rigid judgment criteria for socket-type joints.

Du RJ [29] provided the judgment criteria for the semi-rigid connection joints of socket-type support formwork, as shown in Fig 9.

The curves in the figure were plotted using the following equations.

$$\begin{array}{l} A:M^{*}\leq 2/3,\;M^{*}=25\theta^{*};B:2/3<M^{*}\leq 1,\;M^{*}=(25\theta^{*}+4)/7;C:M^{*}=0.5\theta^{*}.\\ M^{*}=M/M_{\text{p}};\theta^{*}=\theta /\left(M_{\text{P}}l_{\text{b}}/EI_{\text{b}}\right);W_{\text{px}}=\frac{\pi }{4}\left(R^{3}-\frac{r^{4}}{R}\right);I_{\text{b}}=\frac{\pi }{4}(R^{4}-r^{4}), \end{array}$$

where *M*_P_ denotes the full plastic bending moment of the horizontal bar; *M*_p_ = *W*_px_ · *f*_y_, where *W*_px_ denotes the plastic section modulus; *EI*_b_/*l*_b_ denotes the linear stiffness of the horizontal bar; and *I*_b_ denotes the cross-section inertia moment of the horizontal bar.

For disc-buckle type formwork support, the section size of the horizontal bar was set to *R* = 24 mm and *t* = 2.5 mm. According to the material test results, the yield strength of the horizontal bar was set to 401.44 × 10^6^ Pa and the elastic modulus was set to 2.06 × 10^11^ Pa. Therefore, it was calculated that *W*_px_ = 3.8648 × 10^−6^ m^3^; subsequently, it was calculated that *M*_P_ = 1551.5 N·m and *M*_p_*l*_b_/*EI*_b_ = 0.02436 rad.

**Fig 10:**
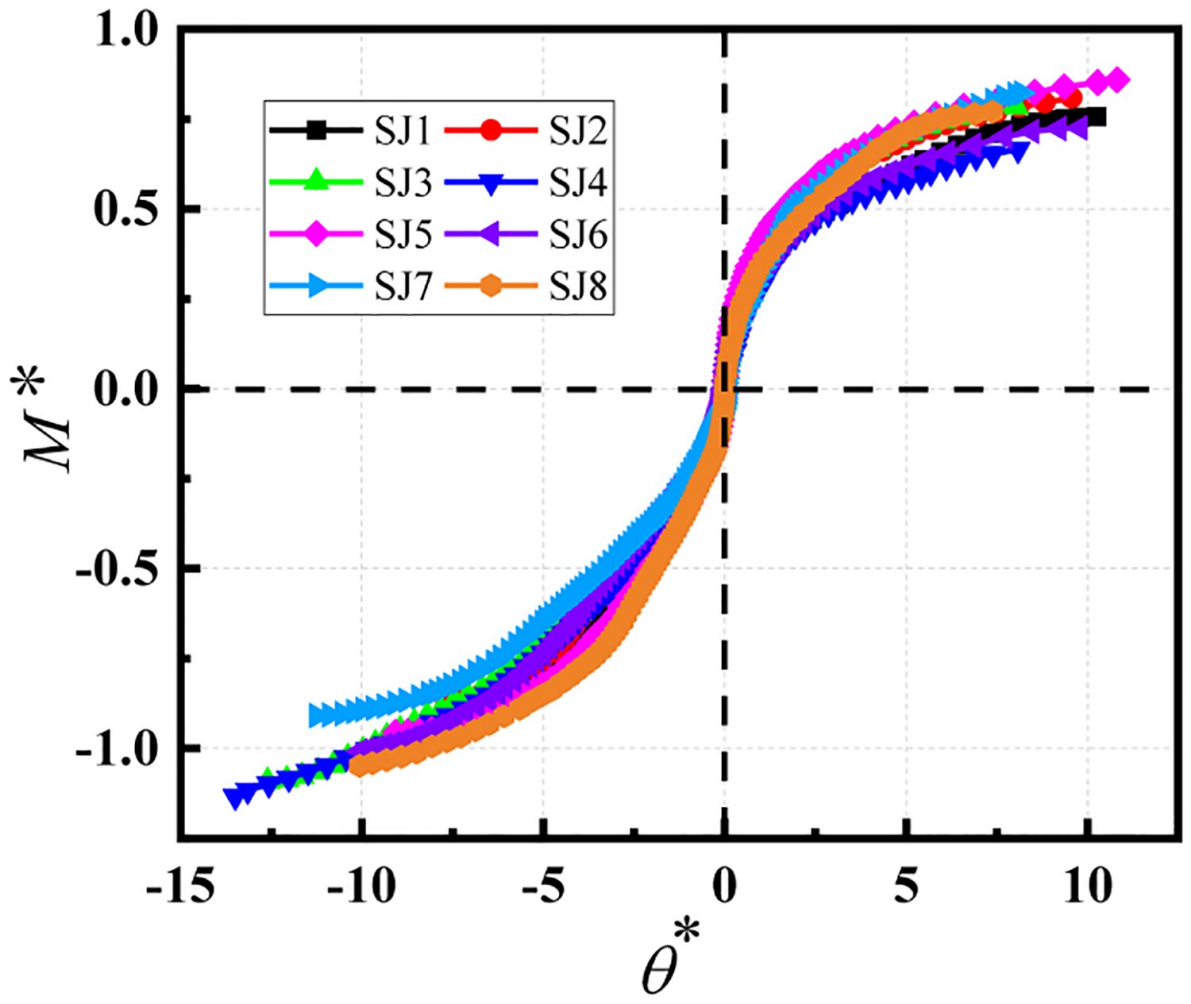
Dimensionless bending moment–rotation curves.

**Fig 11:**
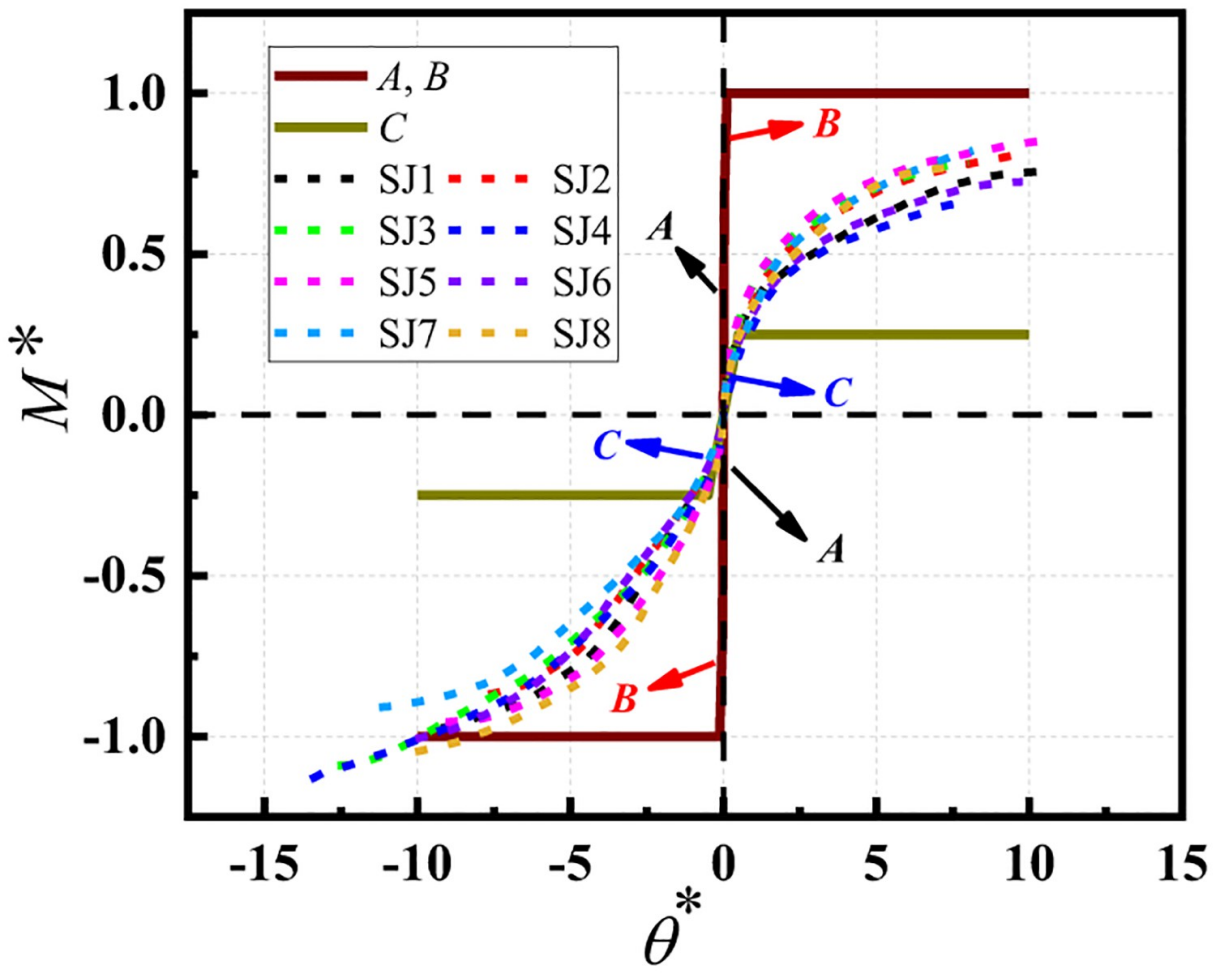
Rigid regions of the experimental joints.

The experimental data of the connection joints were processed without dimensions using the above equation (Fig 10) and corresponding curves were drawn to divide the semi-rigid joint region, as shown in Fig 11.

Fig 11 shows the dimensionless bending moment–rotation curves of the eight groups of joints. From the figure, it can be seen that the connection joints of the disc-buckle type formwork support are typical semi-rigid connection joints. Essentially, the dimensionless bending moment–rotation curves of the connection joints were in the middle of the semi-rigid joint region, regardless of whether the joint was in a positive or negative bending state.

### 3.3 Bending Stiffness value of the semi-rigid joints of the disc-buckle type formwork support

**Fig 12:**
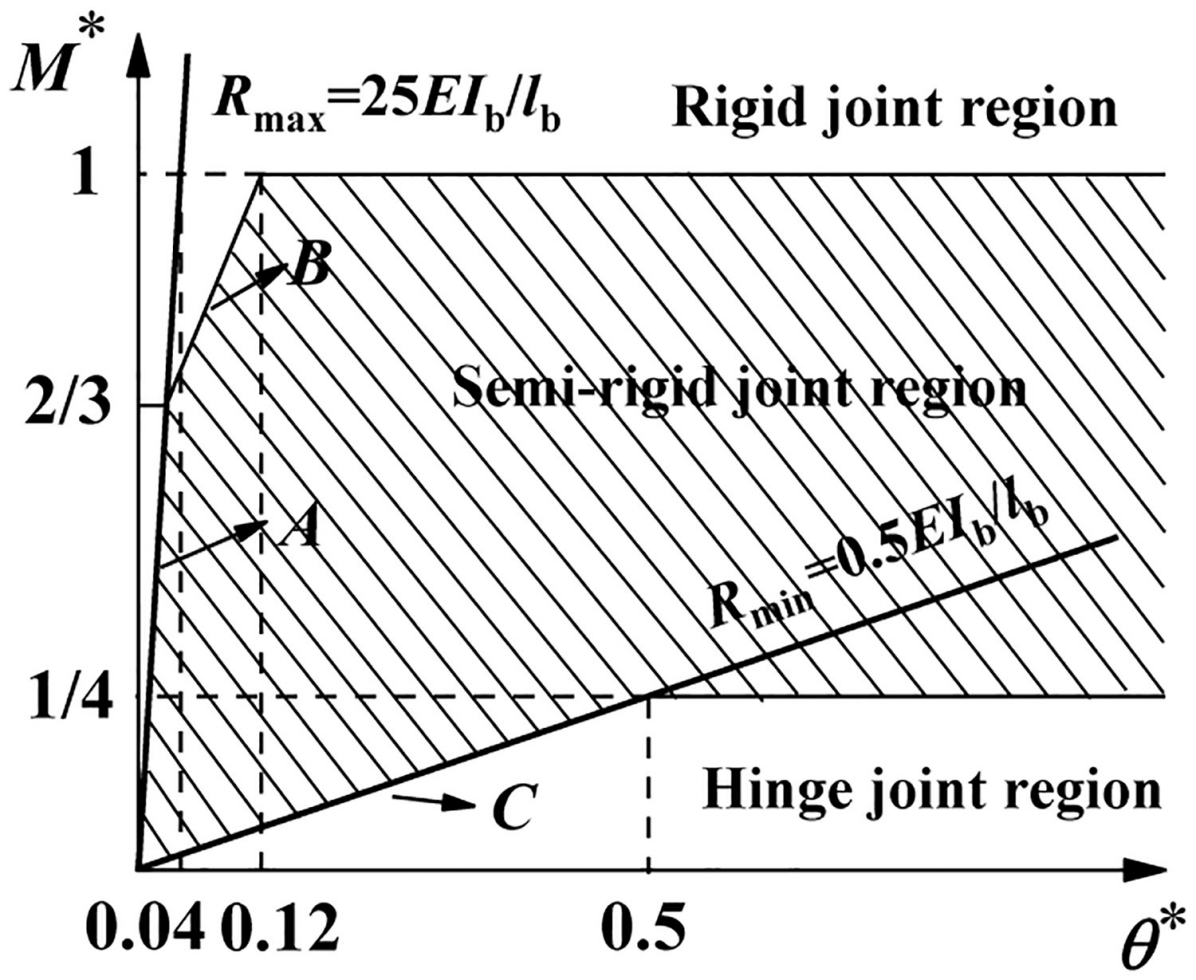
Diagram of initial bending stiffness of socket-type joint.

Du RJ [29] summarized the division region and proposed the judgment criteria of semi-rigid joints of socket-type formwork supports, as shown in Fig 9. According to the above-mentioned division method, the minimum initial joint bending stiffness *R*_min_ of the socket-type formwork support can be derived from M* = 25θ*, which results in *R*_min_ = *M*/*θ* = 0.5*EI*_b_ / *l*_b._ Additionally, the maximum initial joint bending stiffness *R*_max_ can be derived from M* = 25θ*, which results in *R*_min_ = *M*/*θ* = 25*EI*_b_ / *l*_b_, as shown in Fig 12.

As seen from Fig 12, the specifications of the horizontal bars were determined, and *R*_min_ and *R*_max_ were related to the transverse and longitudinal distance, *l*_b_. The results of the range of initial joint bending stiffness of the horizontal bars of the two specifications are as follows.

Condition 1:

when *l*_b,min_ = 0.3m, *R*_min_ = *M*/*θ* = 0.5*EI*_bA_ / *l*_b,min_ = 31.9(kN · m)/rad;
when *l*_b,max_ = 2.0m, *R*_min_ = *M*/*θ* = 0.5*EI*_bA_ / *l*_b,max_ = 4.8(kN · m)/rad;
when *l*_b,min_ = 0.3m, *R*_max_ = *M*/*θ* = 25*EI*_bA_ / *l*_b,min_ = 1593.1(kN · m)/rad;
when *l*_b,max_ = 2.0m, *R*_max_ = *M*/*θ* = 25*EI*_bA_ / *l*_b,max_ = 239.0(kN · m)/rad.

When the transverse and longitudinal distance differed, the regional boundary of initial bending stiffness of the semi-rigid joint differed, as listed in Table 4.

**Table 4:** Regional boundary of initial bending stiffness of the semi-rigid joint with different transverse and longitudinal distances in model of A-SG.

***l***_**b**_ **/m**	0.3	0.6	0.9	1.2	1.5	1.8	2.0
***R***_**min**_ **/(kN·m)/rad**	31.9	15.9	10.6	8.0	6.4	5.3	4.8
***R***_**max**_ **/(kN·m)/rad**	1593.1	796.5	531.0	398.3	318.6	265.5	239.0

Condition 2:

when *l*_b,min_ = 0.3m, *R*_min_ = *M*/*θ* = 0.5*EI*_bB_ / *l*_b,min_ = 20.8(kN · m)/rad;
when *l*_b,max_ = 2.0m, *R*_min_ = *M*/*θ* = 0.5*EI*_bB_ / *l*_b,max_ = 3.1(kN · m)/rad;
when *l*_b,min_ = 0.3m, *R*_max_ = *M*/*θ* = 25*EI*_bB_ / *l*_b,min_ = 1042.0(kN · m)/rad;
when *l*_b,max_ = 2.0m, *R*_max_ = *M*/*θ* = 25*EI*_bB_ / *l*_b,max_ = 156.3(kN · m)/rad.

When the transverse and longitudinal distance differed, the regional boundary of initial bending stiffness of the semi-rigid joint differed, as listed in Table 5.

**Table 5:** Regional boundary of initial bending stiffness of the semi-rigid joint with different transverse and longitudinal distances in model of B-SG.

***l***_**b**_ **/m**	0.3	0.6	0.9	1.2	1.5	1.8	2.0
***R***_**min**_ **/(kN·m)/rad**	20.8	10.4	6.9	5.2	4.2	3.5	3.1
***R***_**max**_ **/(kN·m)/rad**	1042.0	521.0	347.3	260.5	208.4	173.7	156.3

From Tables 4 and 5, it can be inferred that when the transverse and longitudinal distance was determined, the regional boundary and the value range of the effective length correction factor of semi-rigid joints could be determined.

**Fig 13:**
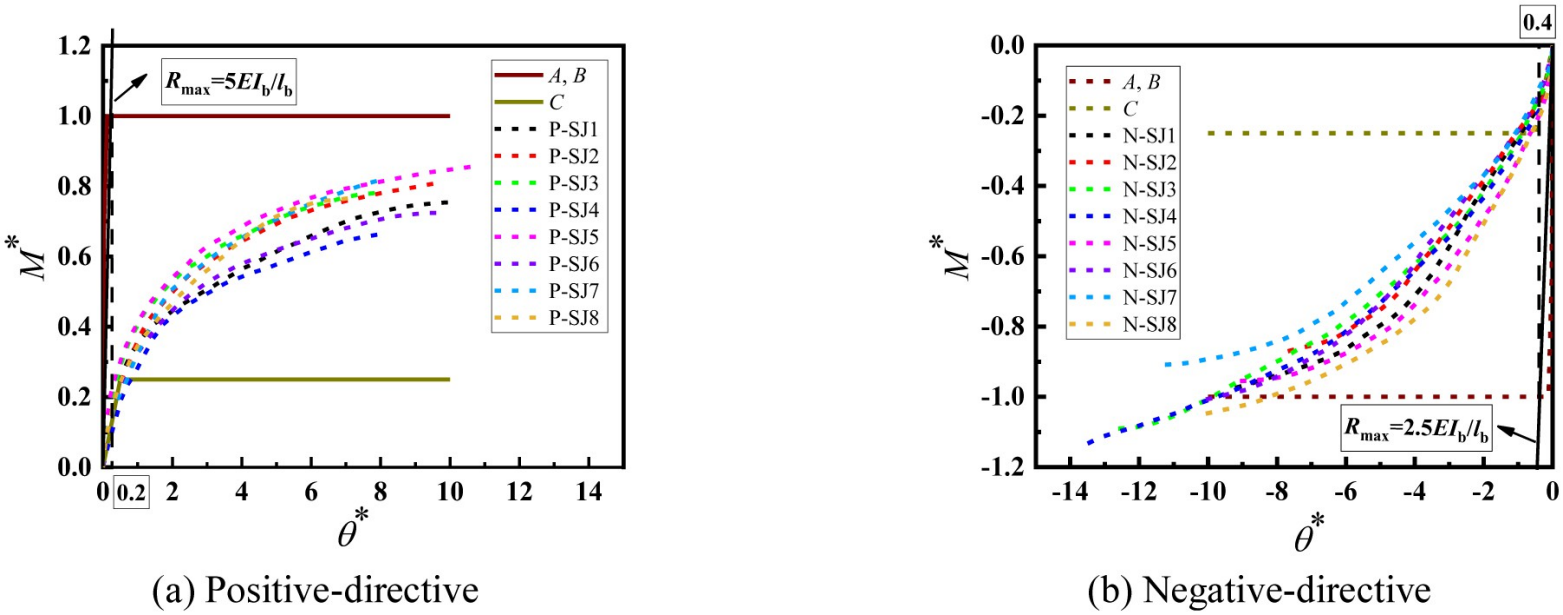
Diagram of initial bending stiffness of disc-buckle type joints. (a) Positive-directive. (b) Negative-directive.

Fig 13 shows the positive- and negative-direction bending stiffness obtained from the results of Section 3.2.3. The horizontal bar used in the experiments was model A-SG and the vertical pole was model B-LG from condition 2.

From this analysis, the following can be determined.

When the joint was in the positive bending state, the maximum initial bending stiffness was, *R*_max_ = *M*/*θ* = 5*EI*_b_ / *l*_b_.

When the joint was in the negative bending state, the maximum initial bending stiffness was, *R*_max_ = *M*/*θ* = 2.5*EI*_b_ / *l*_b_.

Regardless of whether the joint was in the positive or negative bending state, the minimum initial bending stiffness was, *R*_min_ = *M*/*θ* = 0.5*EI*_b_ / *l*_b_.

Models A-SG and B-LG were adopted in horizontal bars and vertical poles, respectively. The regional boundary of initial bending stiffness of semi-rigid joint at different transverse and longitudinal distances are summarized in Tables 6 and 7.

**Table 6:** Regional boundary of initial bending stiffness of semi-rigid joint under the positive bending.

***l***_**b**_ **/m**	0.3	0.6	0.9	1.2	1.5	1.8	2.0
***R***_**min**_ **/(kN·m)/rad**	31.9	15.9	10.6	8.0	6.4	5.3	4.8
***R***_**max**_ **/(kN·m)/rad**	318.6	159.3	106.2	79.7	63.7	53.1	47.8

**Table 7:** Regional boundary of initial bending stiffness of semi-rigid joint under the negative bending.

***l***_**b**_ **/m**	0.3	0.6	0.9	1.2	1.5	1.8	2.0
***R***_**min**_ **/(kN·m)/rad**	31.9	15.9	10.6	8.0	6.4	5.3	4.8
***R***_**max**_ **/(kN·m)/rad**	159.3	79.7	53.1	39.8	31.9	26.6	23.9

## 4 Influence of setting parameters on the effective length correction factor

### 4.1 Influence of setting parameters under different conditions

According to the analysis performed above, the equation of the effective length correction factor is $\mu_{\text{mod}}=\sqrt{\frac{7.5\alpha k\alpha k+4(\alpha k+\alpha k)+1.52}{7.5\alpha k\alpha k+\alpha k+\alpha k}}$. Let *K* = *αk*, where $\alpha =\frac{1}{1+6EI_{\text{b}}/l_{\text{b}}R}=\frac{l_{\text{b}}R}{l_{\text{b}}R+6EI_{\text{b}}}$. Thus, $K=\alpha k=\frac{I_{\text{b}}}{I_{\text{c}}}\times \frac{l_{\text{c}}R}{l_{\text{b}}R+6EI_{\text{b}}}$ and $\mu_{\text{mod}}=\sqrt{\frac{7.5\cdot K^{2}+8\cdot K+1.52}{7.5\cdot K^{2}+2\cdot K}}$.

The effective length correction factor *μ*_mod_ was primarily considered in the four conditions.

Condition 1: model A-SG was adopted in the horizontal bar, $\mu_{\text{mod}\;\text{I}}=\sqrt{\frac{7.5\cdot K_{\text{I}}{}^{2}+8\cdot K_{\text{I}}+1.52}{7.5\cdot K_{\text{I}}{}^{2}+2\cdot K_{\text{I}}}}$;

Condition 2: model A-SG was adopted in the horizontal bar, $\mu_{\text{mod}\;\text{II}}=\sqrt{\frac{7.5\cdot K_{\text{II}}{}^{2}+8\cdot K_{\text{II}}+1.52}{7.5\cdot K_{\text{II}}{}^{2}+2\cdot K_{\text{II}}}}$.

In Conditions 1 and 2, when the joint was in the positive bending state, the value range of the initial bending stiffness was *R*_AP_ = 5 ~ 319(kN · m)/rad. When the joint was in the negative bending state, the value range of the initial bending stiffness was *R*_AN_ = 5 ~ 159(kN · m)/rad.

Condition 3: model B-SG was adopted in the horizontal bar, $\mu_{\text{mod}\;\text{III}}=\sqrt{\frac{7.5\cdot K_{\text{III}}{}^{2}+8\cdot K_{\text{III}}+1.52}{7.5\cdot K_{\text{III}}{}^{2}+2\cdot K_{\text{III}}}}$;

Condition 4: model B-SG was adopted in the horizontal bar, $\mu_{\text{mod}\;\text{IV}}=\sqrt{\frac{7.5\cdot K_{\text{IV}}{}^{2}+8\cdot K_{\text{IV}}+1.52}{7.5\cdot K_{\text{IV}}{}^{2}+2\cdot K_{\text{IV}}}}$.

In Conditions 3 and 4, when the joint was in the positive bending state, the value range of the initial bending stiffness was *R*_BP_ = 3 ~ 208(kN · m)/rad. When the joint was in the negative bending state, the value range of the initial bending stiffness was *R*_BN_ = 3 ~ 104(kN · m)/rad.

**Fig 14:**
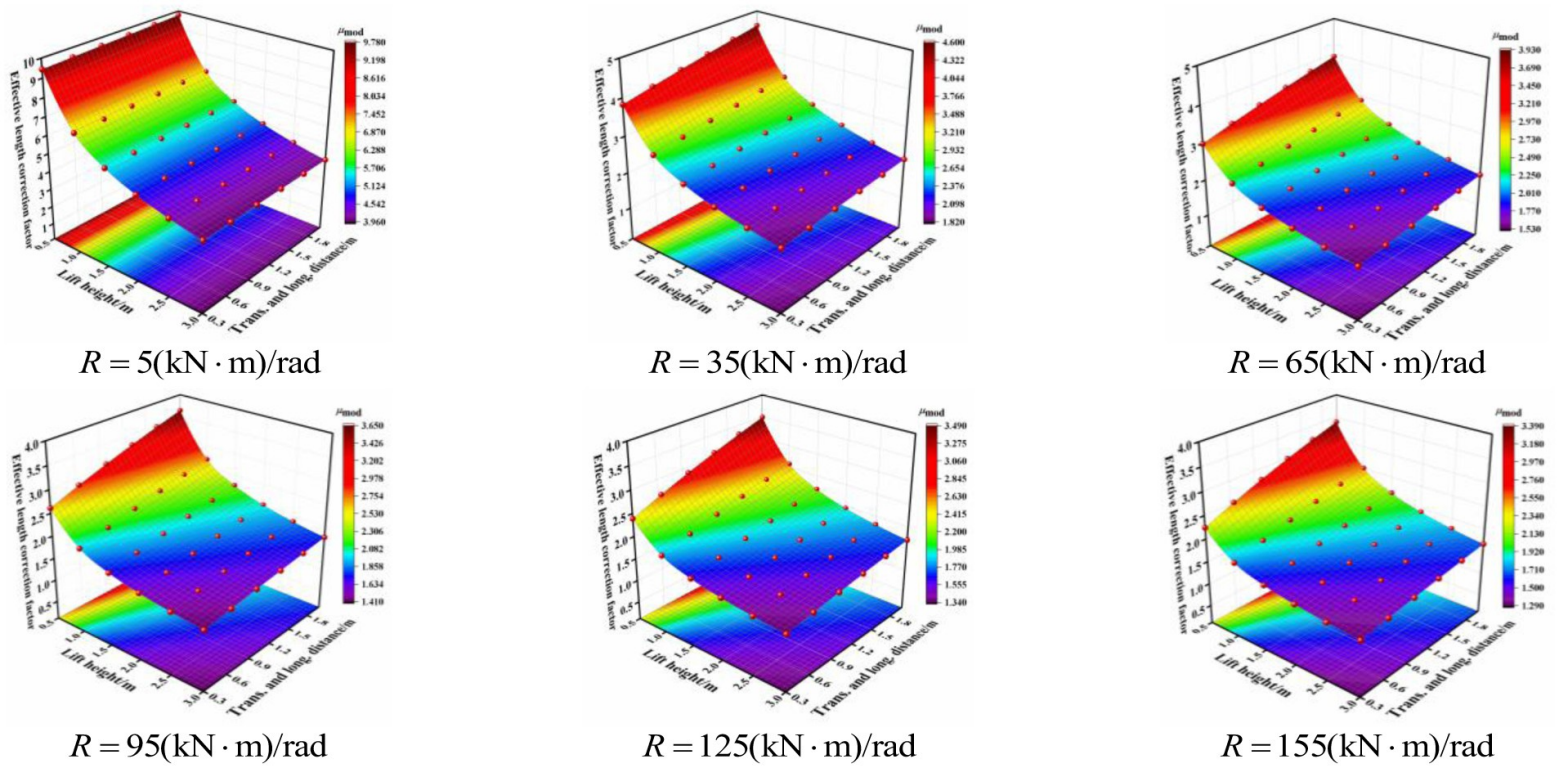
Three-dimensional cloud diagrams of the effective length correction factor under different joint bending stiffness of condition 1.

The effective length correction factor of the aforementioned four conditions was obtained using MATLAB. When considering the influence of the effective length correction factor on the joint bending stiffness, transverse and longitudinal distance, and lift height, the three-dimensional cloud diagram of the respective relationships were obtained, as shown in Fig 14. For all conditions, with an increase in the joint bending stiffness, the influence of the transverse and longitudinal distance on the effective length correction factor was progressively obvious when the lift height was small. The effective length correction factor increased with increasing transverse and longitudinal distance. With an increase in the joint bending stiffness, the influence of the lift height on the effective length correction factor was reduced when the transverse and longitudinal distance was small. The effective length correction factor decreased with increasing lift height.

The results indicate that when the overall high support system bore the initial load, the lift height had a significant influence on the effective length correction factor; this influence was more obvious when the transverse and longitudinal distance was large. Furthermore, the transverse and longitudinal distances affected the effective length correction factor when the lift height was small. When the load of the overall high support system decreased gradually, the lift height played a decisive role in the effective length correction factor and the transverse and longitudinal distances had little influence.

**Fig 15:**
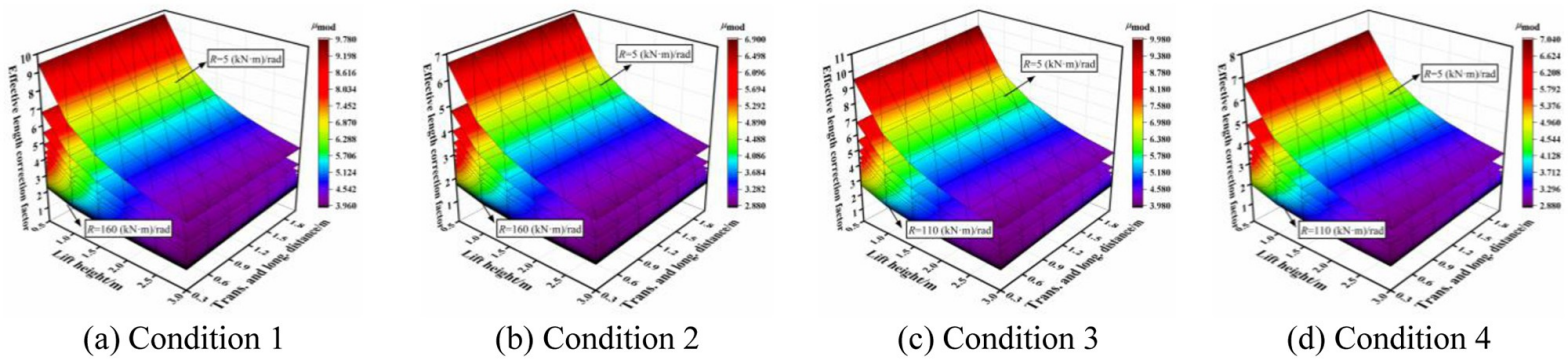
Effective length correction factor under different conditions. (a) Condition 1. (b) Condition 2. (c) Condition 3. (d) Condition 4.

The combined conditions between different types of horizontal bars and vertical poles are shown in Fig 15. When the joint bending stiffness was same for all cases, the effective length correction factor varied from small to large in the order of: Condition 2 < Condition 4 < Condition 1 < Condition 3. With an increase in the joint bending stiffness, the influence of setting parameters on the effective length correction factor was decreased. With a decrease in the joint bending stiffness, condition 2 was the most stable in the entire working state with the minimum effective length correction factor.

Through this process, the bending moment–rotation curves of the joint were determined, and subsequently, the joint bending stiffness was obtained. After the setting parameters were determined, the effective length correction factor was calculated; note that herein, the value of the effective length correction factor was taken during the stiffness change to accurately control the process description of the overall stability bearing capacity.

### 4.2 Determination of value area of the effective length correction factor under different conditions

**Fig 16:**
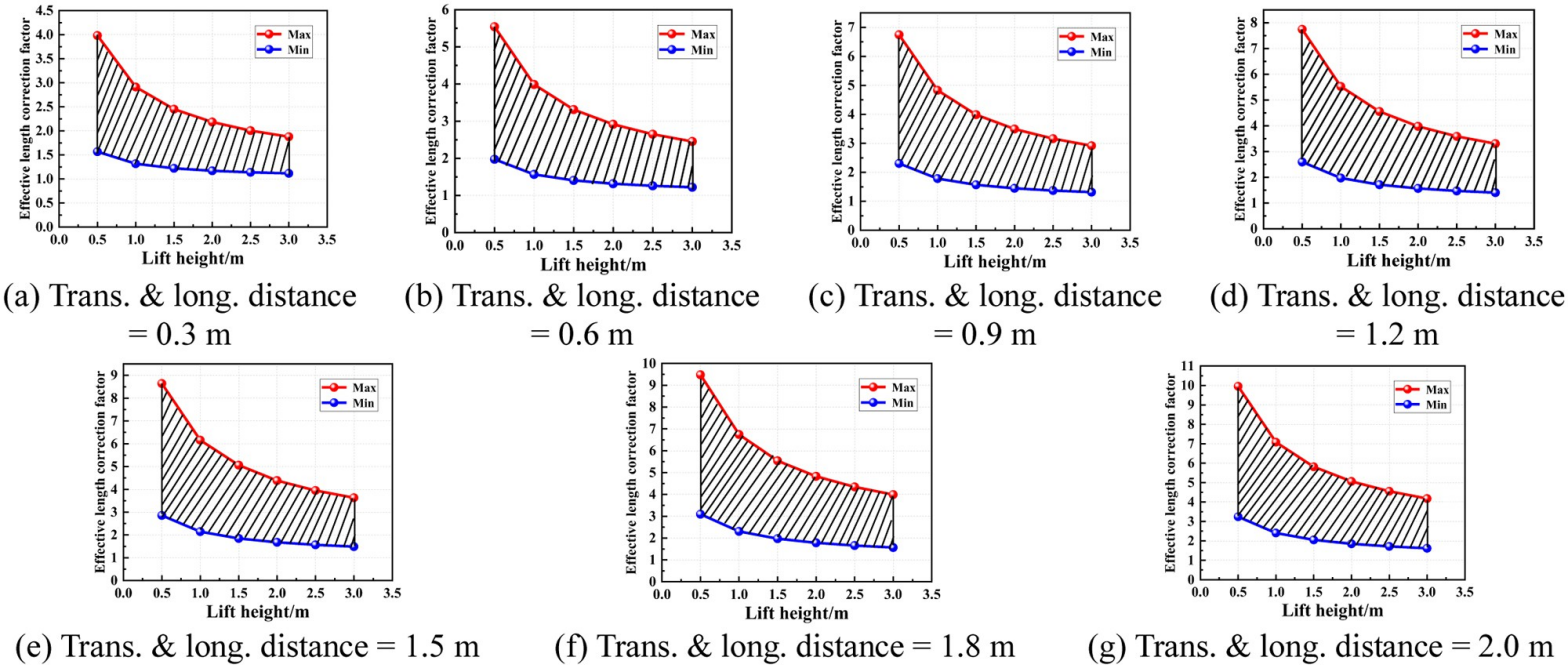
Value areas of the effective length correction factor of condition 1. (a) Trans. & long. distance = 0.3 m. (b) Trans. & long. distance = 0.6 m. (c) Trans. & long. distance = 0.9 m. (d) Trans. & long. distance = 1.2 m. (e) Trans. & long. distance = 1.5 m. (f) Trans. & long. distance = 1.8 m. (g) Trans. & long. distance = 2.0 m.

**Fig 17:**
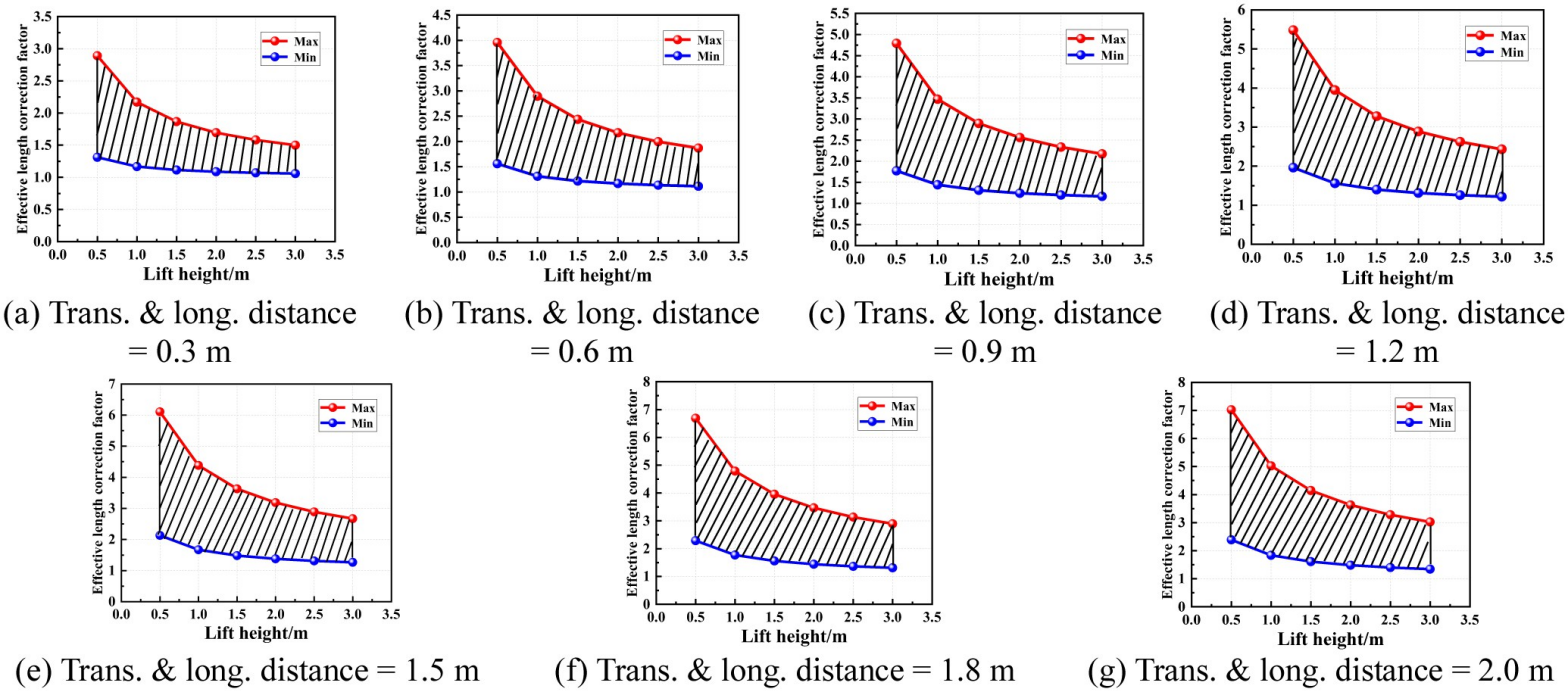
Value areas of the effective length correction factor of condition 2. (a) Trans. & long. distance = 0.3 m. (b) Trans. & long. distance = 0.6 m. (c) Trans. & long. distance = 0.9 m. (d) Trans. & long. distance = 1.2 m. (e) Trans. & long. distance = 1.5 m. (f) Trans. & long. distance = 1.8 m. (g) Trans. & long. distance = 2.0 m.

**Fig 18:**
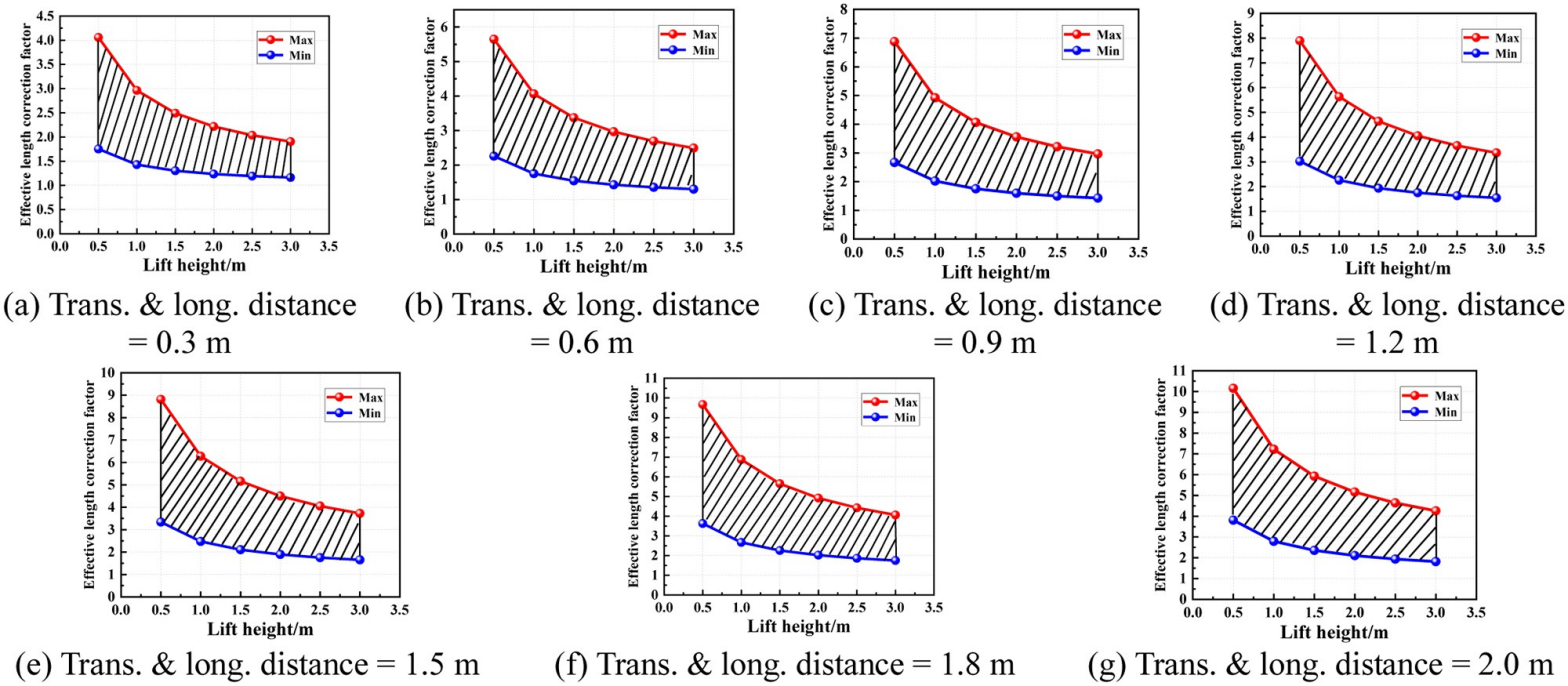
Value areas of the effective length correction factor of condition 3. (a) Trans. & long. distance = 0.3 m. (b) Trans. & long. distance = 0.6 m. (c) Trans. & long. distance = 0.9 m. (d) Trans. & long. distance = 1.2 m. (e) Trans. & long. distance = 1.5 m. (f) Trans. & long. distance = 1.8 m. (g) Trans. & long. distance = 2.0 m.

**Fig 19:**
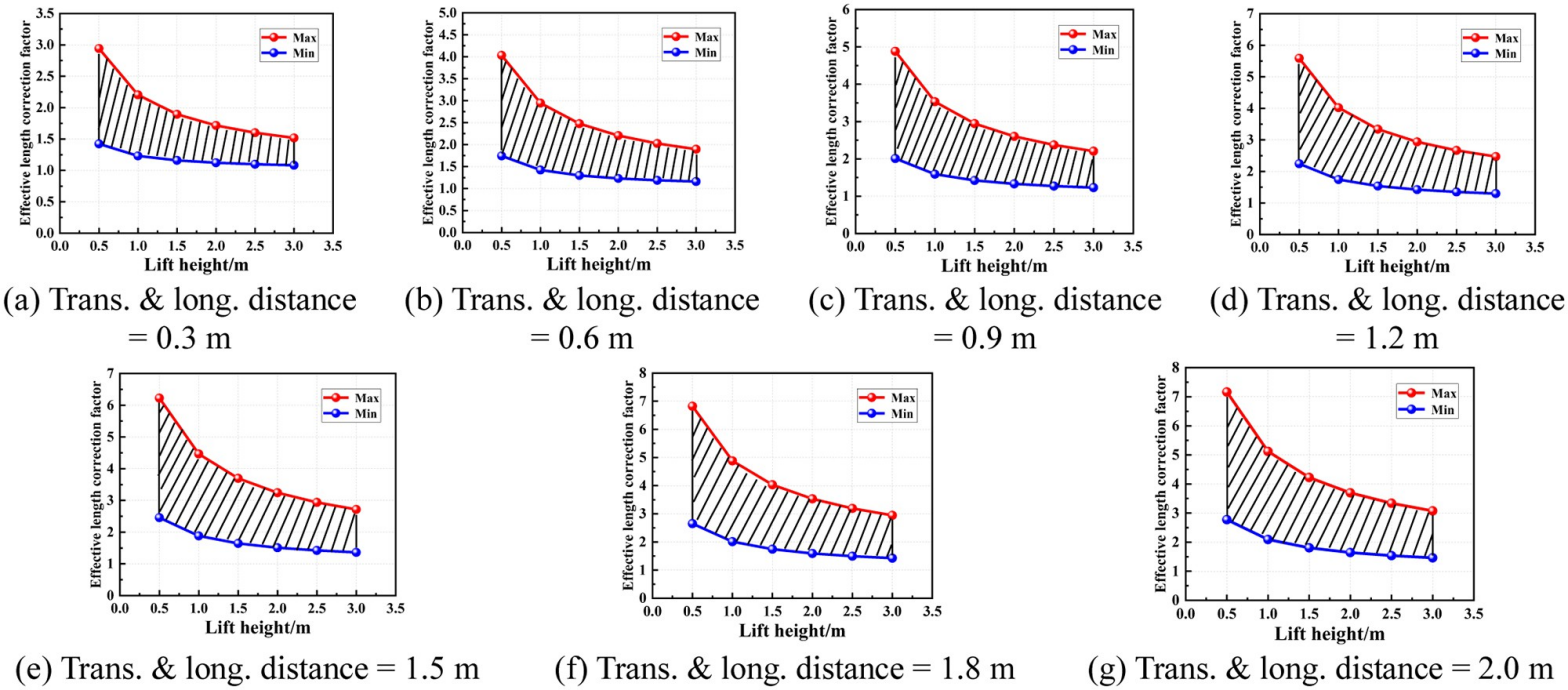
Value areas of the effective length correction factor of condition 4. (a) Trans. & long. distance = 0.3 m. (b) Trans. & long. Distance = 0.6 m. (c) Trans. & long. distance = 0.9 m. (d) Trans. & long. distance = 1.2 m. (e) Trans. & long. distance = 1.5 m. (f) Trans. & long. Distance = 1.8 m. (g) Trans. & long. distance = 2.0 m.

According to the region of the semi-rigid joint bending stiffness of the socket-type formwork support, the value area of the effective length correction factor under various conditions was determined. Figs 16–19 show the value areas of the effective length correction factor corresponding to various values of lift height in different transverse and longitudinal distances.

### 4.3 Value of the effective length under different conditions

**Fig 20:**
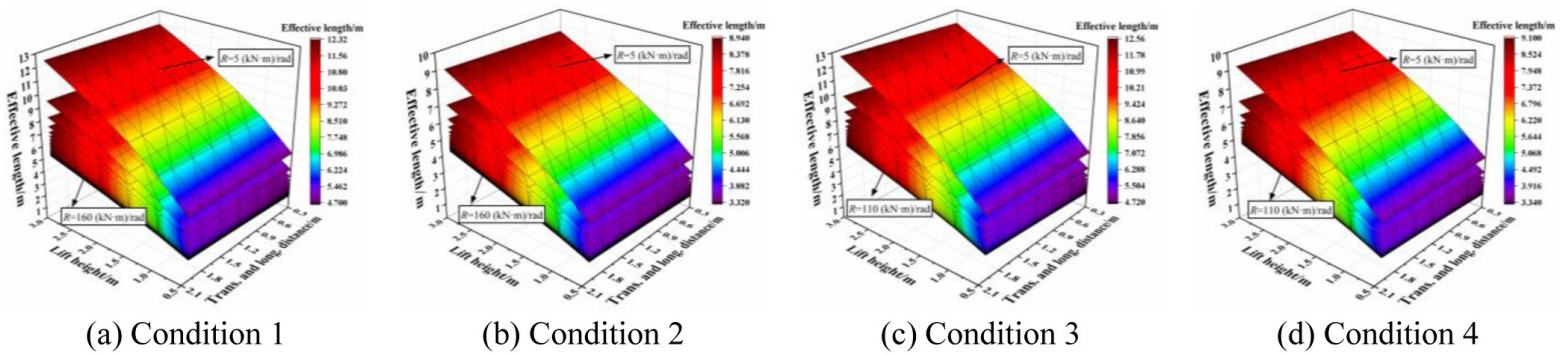
Value of the effective length under different conditions. (a) Condition 1. (b) Condition 2. (c) Condition 3. (d) Condition 4.

The effective length was obtained by multiplying the effective length correction factor of the four conditions in Fig 20 and the length of the vertical pole. Fig 20 shows that the effective length of the four conditions decreases with increase in the joint bending stiffness; essentially, the smaller the joint bending stiffness, the greater the influence of the effective length. Note that the effective length did not change when the joint bending stiffness was sufficiently large. The effective length varied as: Condition 2 < Condition 4 < Condition 1 < Condition 3. This indicates that Condition 2 was the best condition for stability.

## 5 Influence of joint bending stiffness on the effective length correction factor

**Fig 21:**
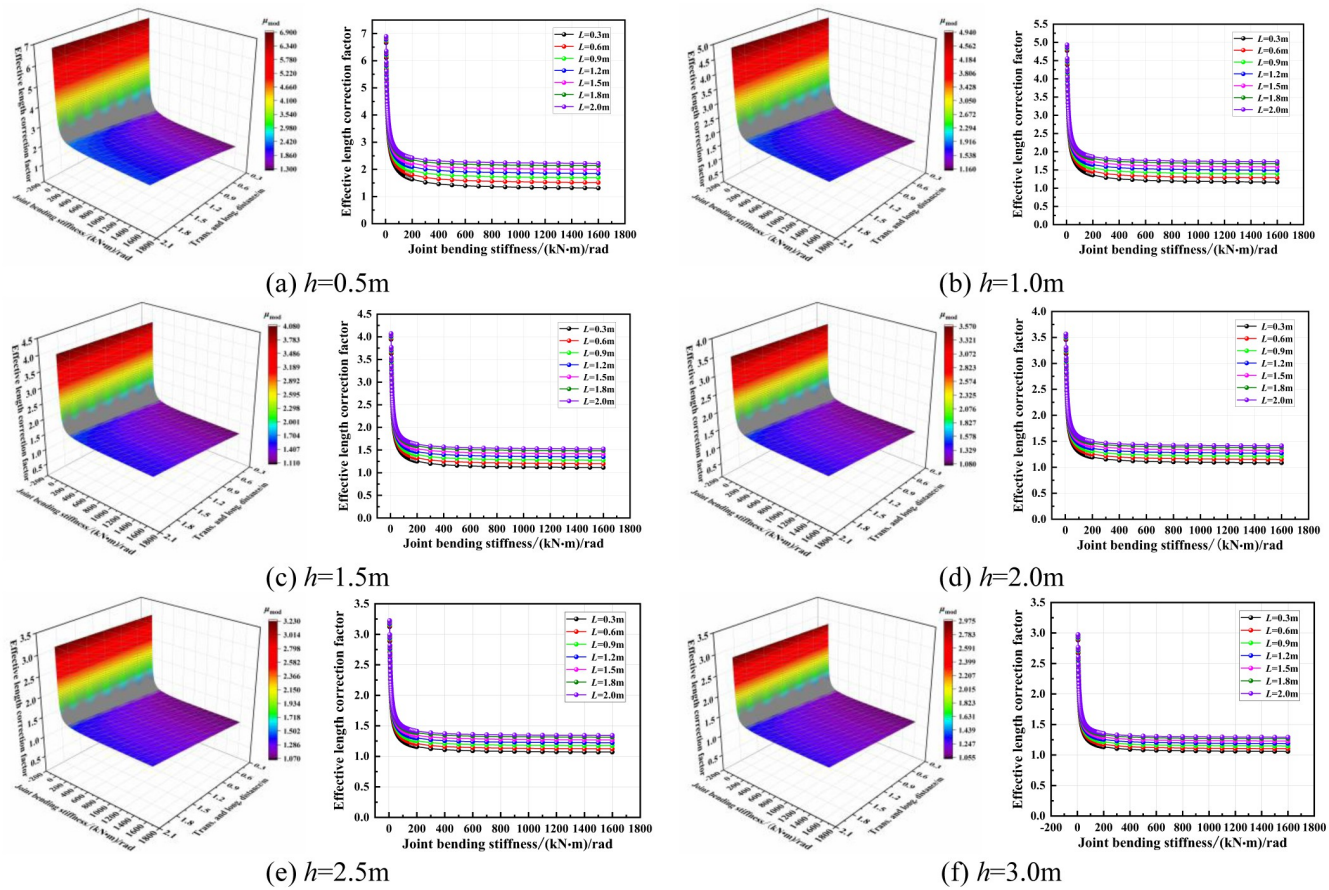
Three-dimensional diagrams of the influence of the joint bending stiffness on the effective length correction factor. (a) *h* = 0.5m. (b) *h* = 1.0m. (c) *h* = 1.5m. (d) *h* = 2.0m. (e) *h* = 2.5m. (f) *h* = 3.0m.

The influence of the joint bending stiffness on the effective length correction factor when the lift height was varied is shown in Fig 21. As seen from the figure, when the joint bending stiffness was less than 100 (kN·m)/rad, the effective length correction factor was significantly affected by the joint bending stiffness. With increase in the joint bending stiffness, the effective length correction factor decreased rapidly and the transverse and longitudinal distance had no influence. When the joint bending stiffness was increased to greater than 100 (kN·m)/rad, the effective length correction factor remained unchanged and the transverse and longitudinal distance had little influence. Essentially, the smaller the joint bending stiffness, the more obvious the influence of the lift height on the effective length correction factor. When the joint bending stiffness was increased to greater than 200 (kN·m)/rad, the influence of lift height on the effective length correction factor was small and unchanged.

According to the above research, the joint initial bending stiffness *R* = 5~319 (kN·m)/rad in condition 1 and condition 2, so the bending stiffness of the relative dangerous condition is set to *R* = 150 (kN·m)/rad, and the effective length correction factor can be obtained. Similarly, the joint initial bending stiffness *R* = 3~208 (kN·m)/rad in condition 3 and condition 4, so the bending stiffness of the relative dangerous condition is set to *R* = 100 (kN·m)/rad.

## 6 Influence of geometrical size on the effective length correction factor

### 6.1 Influence of section size of horizontal bar

#### 6.1.1 Influence of outer diameter size of horizontal bar section

To study the influence of the section size of horizontal bar on the effective length correction factor, the outer diameter and wall thickness size of the horizontal bar were set to 48 mm ± 0.5 mm and 2.5mm ± 0.5 mm, respectively. The relevant parameters are listed in Table 8. The section size of the vertical pole’s influence on the relationship between the section size of the horizontal bar and effective length correction factor was determined by considering five conditions, namely *ϕ*42 × 3.2, *ϕ*52 × 3.2, *ϕ*48 × 2.6, *ϕ*48 × 3.2, and *ϕ*48 × 3.6.

**Table 8:** Parameters related to section size of horizontal bar and the effective length correction factor.

No.	Outer diameter size of horizontal bar section/mm	Cross sectional moment of inertia of horizontal bar *I*_b_ /cm^4^	6*EI*_b_ / kN·m^2^	Wall thickness size of horizontal bar section/mm	Cross sectional moment of inertia of horizontal bar *I*_b_ /cm^4^	6*EI*_b_ / kN·m^2^	*I*_c_ /cm^4^
**1**	*ϕ*43 × 2.5	6.55	80.9580	*ϕ*48 × 2.0	7.66	94.6776	*ϕ*42 × 3.2 = 7.39 *ϕ*52 × 3.2 = 14.67 *ϕ*48 × 2.6 = 9.59 *ϕ*48 × 3.2 = 11.36 *ϕ*48 × 3.6 = 12.46
**2**	*ϕ*44 × 2.5	7.04	87.0144	*ϕ*48 × 2.1	7.99	98.7564	
**3**	*ϕ*45 × 2.5	7.56	93.4416	*ϕ*48 × 2.2	8.32	102.8352	
**4**	*ϕ*46 × 2.5	8.11	100.2396	*ϕ*48 × 2.3	8.64	106.7904	
**5**	*ϕ*47 × 2.5	8.68	107.2848	*ϕ*48 × 2.4	8.96	110.7456	
**6**	*ϕ*48 × 2.5	9.28	114.7008	*ϕ*48 × 2.5	9.28	114.7008	
**7**	*ϕ*49 × 2.5	9.90	122.3640	*ϕ*48 × 2.6	9.59	118.5324	
**8**	*ϕ*50 × 2.5	10.55	130.3980	*ϕ*48 × 2.7	9.89	122.2404	
**9**	*ϕ*51 × 2.5	11.23	138.8028	*ϕ*48 × 2.8	10.19	125.9484	
**10**	*ϕ*52 × 2.5	11.94	147.5784	*ϕ*48 × 2.9	10.49	129.6564	
**11**	*ϕ*53 × 2.5	12.67	156.6012	*ϕ*48 × 3.0	10.78	133.2408	

**Fig 22:**
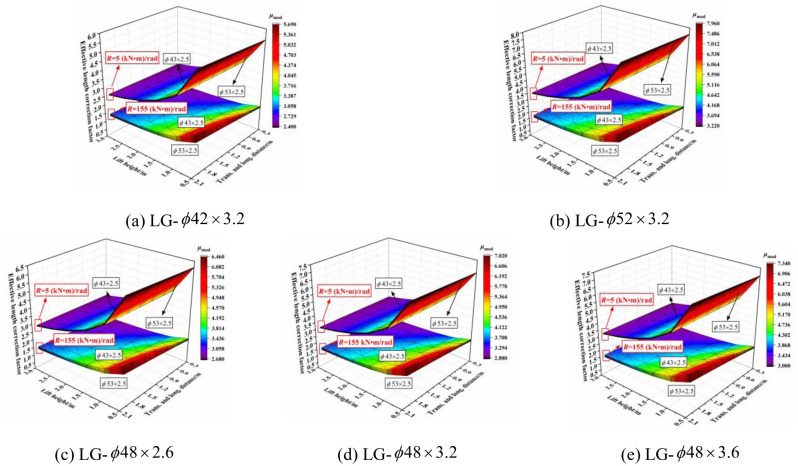
Three-dimensional diagrams of the influence of the outer diameter size of the horizontal bar section on the effective length correction factor. (a) LG-*ϕ*42 × 3.2. (b) LG-*ϕ*52 × 3.2. (c) LG-*ϕ*48 × 2.6. (d) LG-*ϕ*48 × 3.2. (e) LG-*ϕ*48 × 3.6.

The three-dimensional relationship between the outer diameter size of the horizontal bar section and effective length correction factor when the section size of the vertical pole was varied is shown in Fig 22. When the joint bending stiffness was large, the larger the transverse and longitudinal distance was, the smaller the lift height was; furthermore, the influence of the outer diameter size of the horizontal bar section on the effective length correction factor was more obvious. The effective length correction factor decreased with increasing outer diameter size of the horizontal bar section. When the transverse and longitudinal distance was set to 0.3 m, the outer diameter size of the horizontal bar section had no influence on the effective length correction factor. The influence decreased with decreasing joint bending stiffness, and finally, had little influence. This rule was not affected by the size of the transverse and longitudinal distances, or lift height. Essentially, the influence was independent of the section size of the vertical pole.

#### 6.1.2 Influence of wall thickness size of horizontal bar section

**Fig 23:**
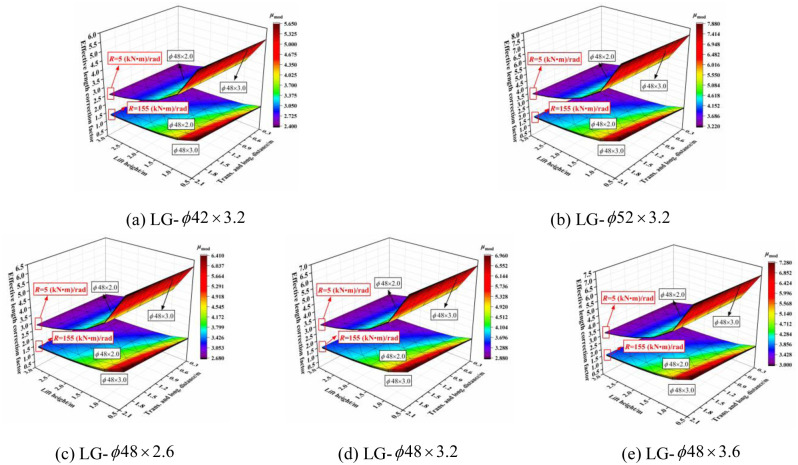
Three-dimensional diagrams of the influence of the wall thickness size of the horizontal bar section on the effective length correction factor. (a) LG-*ϕ*42 × 3.2. (b) LG-*ϕ*52 × 3.2. (c) LG-*ϕ*48 × 2.6. (d) LG-*ϕ*48 × 3.2. (e) LG-*ϕ*48 × 3.6.

The three-dimensional relationship between the wall thickness size of the horizontal bar section and effective length correction factor when the section size of the vertical pole was varied is shown in Fig 23. When the joint bending stiffness was large, the wall thickness had no influence on the effective length correction factor, but with increasing transverse and longitudinal distances and decreasing lift height, the influence gradually increased. The influence of the wall thickness size decreased with decreasing joint bending stiffness, until finally, it had no influence. This rule was not affected by the size of the transverse and longitudinal distances or lift height.

With increasing outer diameter and wall thickness of the horizontal bar section, the effective length correction factor exhibited a decreasing trend, but the overall effect was small. There was only an influence when the joint bending stiffness was large, the transverse and longitudinal distance was set to 2.0 m, and the lift height was set to 0.5 m. This indicates that the section size of the horizontal bar had an influence on the effective length correction factor under the initial joint bending stiffness when the transverse and longitudinal distance was large or the lift height was small in the setting parameters, and the outer diameter had a significant influence on the effective length correction factor. Essentially, the larger the section size of the horizontal bar, the smaller the effective length correction factor, and the more conducive it is to the overall stability.

### 6.2 Influence of section size of vertical pole

#### 6.2.1 Influence of outer diameter size of vertical pole section

To study the influence of the section size of the vertical pole on the effective length correction factor, the section size selected was relatively large; the outer diameter and wall thickness of the vertical pole were set to 48 + 0.4 mm and 48–0.6 mm, respectively. The relevant parameters are listed in Table 9. The influence of the section size of the horizontal bar on the relationship between the section size of the vertical pole and the effective length correction factor was determined by considering five conditions, namely *ϕ*43 × 2.5, *ϕ*53 × 2.5, *ϕ*48 × 2.0, *ϕ*48 × 2.5, and *ϕ*48 × 3.0.

**Table 9:** Parameters related to section size of vertical pole and the effective length correction factor.

No.	Outer diameter size of vertical pole section/mm	Cross sectional moment of inertia of vertical pole *I*_c_ /cm^4^	6*EI*_b_/(kN·m^2^)	Wall thickness size of vertical pole section/mm	Cross sectional moment of inertia of vertical pole *I*_c_ /cm^4^	6*EI*_b_/(kN·m^2^)
**1**	*ϕ*42 × 3.2	7.39	*ϕ*43 × 2.5 = 80.958 *ϕ*53 × 2.5 = 156.6012 *ϕ*48 × 2.0 = 94.6776 *ϕ*48 × 2.5 = 114.7008 *ϕ*48 × 3.0 = 133.2408	*ϕ*48 × 2.6	9.59	*ϕ*43 × 2.5 = 80.958 *ϕ*53 × 2.5 = 156.6012 *ϕ*48 × 2.0 = 94.6776 *ϕ*48 × 2.5 = 114.7008 *ϕ*48 × 3.0 = 133.2408
**2**	*ϕ*43 × 3.2	7.97		*ϕ*48 × 2.7	9.89	
**3**	*ϕ*44 × 3.2	8.59		*ϕ*48 × 2.8	10.19	
**4**	*ϕ*45 × 3.2	9.23		*ϕ*48 × 2.9	10.49	
**5**	*ϕ*46 × 3.2	9.91		*ϕ*48 × 3.0	10.78	
**6**	*ϕ*47 × 3.2	10.62		*ϕ*48 × 3.1	11.07	
**7**	*ϕ*48 × 3.2	11.36		*ϕ*48 × 3.2	11.36	
**8**	*ϕ*49 × 3.2	12.13		*ϕ*48 × 3.3	11.64	
**9**	*ϕ*50 × 3.2	12.94		*ϕ*48 × 3.4	11.91	
**10**	*ϕ*51 × 3.2	13.79		*ϕ*48 × 3.5	12.19	
**11**	*ϕ*52 × 3.2	14.67		*ϕ*48 × 3.6	12.46	

**Fig 24:**
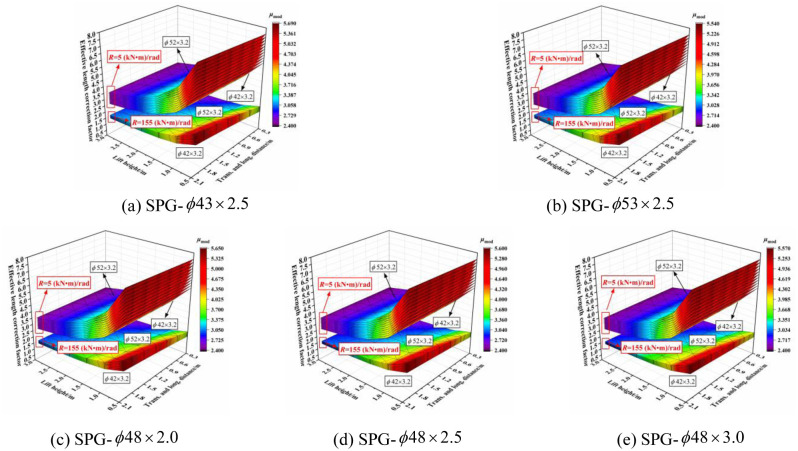
Three-dimensional diagrams of the influence of outer diameter size of vertical pole section on the effective length correction factor. (a) SPG-*ϕ*43 × 2.5. (b) SPG-*ϕ*53 × 2.5. (c) SPG-*ϕ*48 × 2.0. (d) SPG-*ϕ*48 × 2.5. (e) SPG-*ϕ*48 × 3.0.

The three-dimensional relationship between the outer diameter size of the vertical pole section and effective length correction factor when the section size of the horizontal bar was varied is shown in Fig 24. The effective length correction factor increased with increasing diameter size of the vertical pole section, especially when the joint bending stiffness was small. When the joint bending stiffness was large, the influence was affected by the size of the transverse and longitudinal distance and the lift height. When the transverse and longitudinal distances were small and the lift height was large, the influence was small, and vice versa. When the lift height was set to 0.5 m and the transverse and longitudinal distance was set to 2.0 m, the influence was the largest. With decreasing joint bending stiffness, the influence was progressively larger and exhibited a linear growth trend. The influence of the outer diameter size of the vertical pole section on the effective length correction factor was independent of the section size of the horizontal bar.

#### 6.2.2 Influence of wall thickness size of vertical pole section

**Fig 25:**
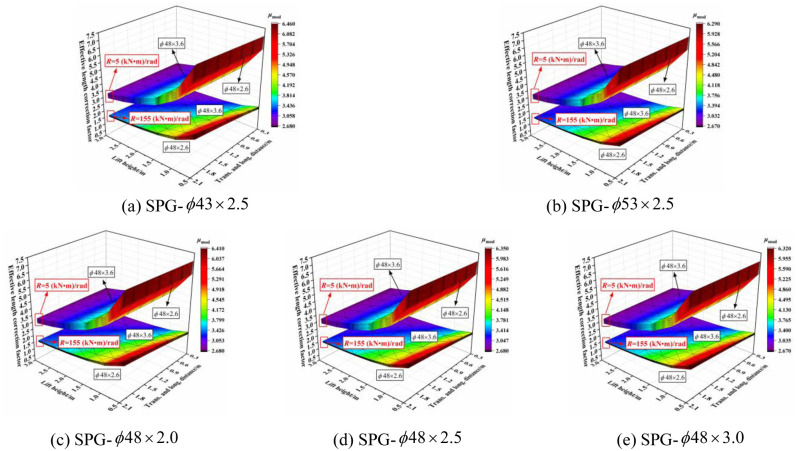
Three-dimensional diagrams of the influence of wall thickness size of vertical pole section on the effective length correction factor. (a) SPG-*ϕ*43 × 2.5. (b) SPG-*ϕ*53 × 2.5. (c) SPG-*ϕ*48 × 2.0. (d) SPG-*ϕ*48 × 2.5. (e) SPG-*ϕ*48 × 3.0.

The three-dimensional relationship between the wall thickness size of the vertical pole section and effective length correction factor when the section size of the horizontal bar was varied is shown in Fig 25. The effective length correction factor increased with increasing wall thickness of the vertical section. When the joint bending stiffness was large, the wall thickness had no influence on the effective length correction factor, but with increasing transverse and longitudinal distance and decreasing lift height, the influence increased gradually. Furthermore, the influence increased slightly and linearly with decreasing joint bending stiffness. Essentially, the influence was independent of the section size of the vertical pole.

In summary, the effective length correction factor increased with increasing section size of the vertical pole. When the joint bending stiffness was small, the influence was more obvious. Whereas, when the joint bending stiffness was large, the transverse and longitudinal distance and lift height had an influence on the relationship between the section size of the vertical pole and effective length correction factor. The outer diameter size of the vertical pole had a significant influence on the effective length correction factor with a small joint bending stiffness, and the influence degree was not affected by the size of the setting parameters.

**Fig 26:**
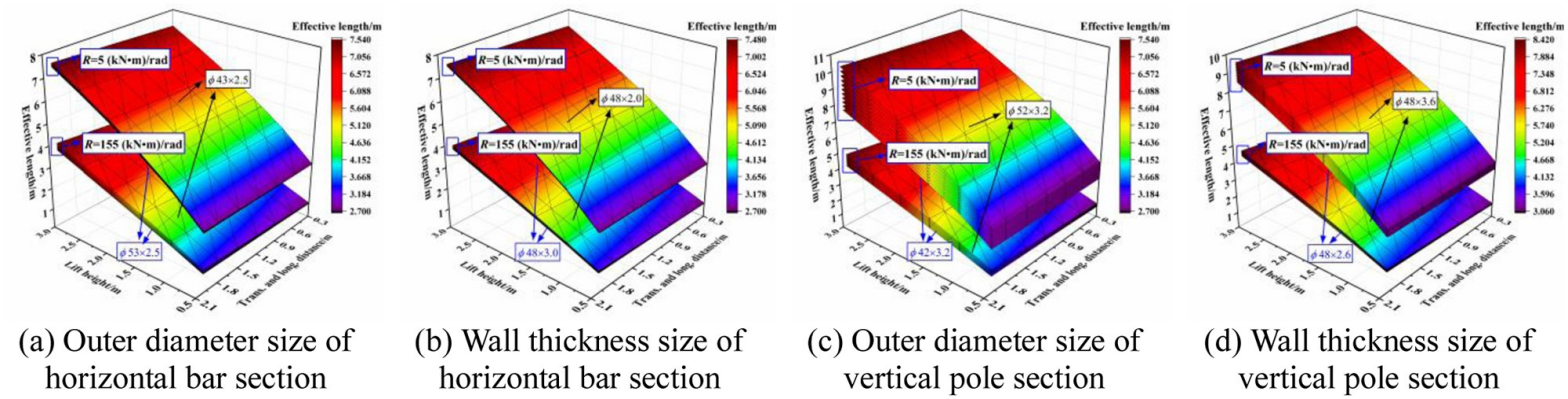
Three-dimensional diagrams of the influence of section size on the effective length. (a) Outer diameter size of horizontal bar section. (b) Wall thickness size of horizontal bar section. (c) Outer diameter size of vertical pole section. (d) Wall thickness size of vertical pole section.

The variation of the effective length with the section size is shown in Fig 26. When the joint bending stiffness was large, i.e., at the initial stage of the overall structure under loading, the outer diameter size of the horizontal bar and the vertical pole section influenced the effective length. The wall thickness size of the section had little influence, and the larger outer diameter size of the horizontal bar section was more conducive to the overall stability. When the joint bending stiffness was small, i.e., the overall structure is in a failure state, the section size of the vertical pole influenced the effective length, the section size of the horizontal bar had little influence, and the effective length increased with increasing section size of the vertical pole.

## 7 Influence of elastic modulus on the effective length correction factor

The influence of elastic modulus on the effective length correction factor was studied by analyzing different sizes of the horizontal bar section and vertical pole section. Five elastic modulus parameters were considered, namely 1.90 × 10^5^N/mm^2^, 2.00 × 10^5^N/mm^2^, 2.06 × 10^5^N/mm^2^, 2.10 × 10^5^N/mm^2^, and 2.20 × 10^5^N/mm^2^. The influence of the section size of the horizontal bar was considered when the specification of the vertical pole was set to *ϕ*48 × 3.2, and the section size of the horizontal bar was set to GK-1: *ϕ*48 × 2.5; GK-2: *ϕ*48 × 2.0; GK-3: *ϕ*48 × 3.0; GK-4: *ϕ*43 × 2.5; and GK-5: *ϕ*53 × 2.5, as summarized in Table 10. The influence of the section size of the vertical pole was considered when the specification of the horizontal bar was set to *ϕ*48 × 2.5, and the section size of the vertical pole was set to GK-6: *ϕ*48 × 2.6; GK-7: *ϕ*48 × 3.6; GK-8: *ϕ*42 × 3.2; and GK-9: *ϕ*52 × 3.2, as summarized in Table 11.

**Table 10:** Influence parameters of elastic modulus of vertical pole with specification *ϕ*48×3.2.

Condition	Specification of horizontal bar /mm	*I*_b_/cm^4^	*E*/(N/mm^2^)	6*EI*_b_/(kN·m^2^)
**GK-1**	*ϕ*48 × 2.5	9.28	1.90×10^5^	105.7920
			2.00×10^5^	111.3600
			2.06×10^5^	114.7008
			2.10×10^5^	116.9280
			2.20×10^5^	122.4960
**GK-2**	*ϕ*48 × 2.0	7.66	1.90×10^5^	87.3240
			2.00×10^5^	91.9200
			2.06×10^5^	94.6776
			2.10×10^5^	96.5160
			2.20×10^5^	101.1120
**GK-3**	*ϕ*48 × 3.0	10.78	1.90×10^5^	122.8920
			2.00×10^5^	129.3600
			2.06×10^5^	133.2408
			2.10×10^5^	135.8280
			2.20×10^5^	142.2960
**GK-4**	*ϕ*43 × 2.5	6.55	1.90×10^5^	74.6700
			2.00×10^5^	78.6000
			2.06×10^5^	80.9580
			2.10×10^5^	82.5300
			2.20×10^5^	86.4600
**GK-5**	*ϕ*53 × 2.5	12.67	1.90×10^5^	144.4380
			2.00×10^5^	152.0400
			2.06×10^5^	156.6012
			2.10×10^5^	159.6420
			2.20×10^5^	167.2440

**Table 11:** Influence parameters of elastic modulus of horizontal bar with specification *ϕ*48×2.5.

Condition	Specification of vertical pole /mm	*I*_c_/ cm^4^	*E*/(N/mm^2^)	6*EI*_b_/(kN·m^2^)
**GK-6**	*ϕ*48 × 2.6	9.59	1.90×10^5^	105.7920
			2.00×10^5^	111.3600
			2.06×10^5^	114.7008
			2.10×10^5^	116.9280
			2.20×10^5^	122.4960
**GK-7**	*ϕ*48 × 3.6	12.46	1.90×10^5^	105.7920
			2.00×10^5^	111.3600
			2.06×10^5^	114.7008
			2.10×10^5^	116.9280
			2.20×10^5^	122.4960
**GK-8**	*ϕ*42 × 3.2	7.39	1.90×10^5^	105.7920
			2.00×10^5^	111.3600
			2.06×10^5^	114.7008
			2.10×10^5^	116.9280
			2.20×10^5^	122.4960
**GK-9**	*ϕ*52 × 3.2	14.67	1.90×10^5^	105.7920
			2.00×10^5^	111.3600
			2.06×10^5^	114.7008
			2.10×10^5^	116.9280
			2.20×10^5^	122.4960

**Fig 27:**
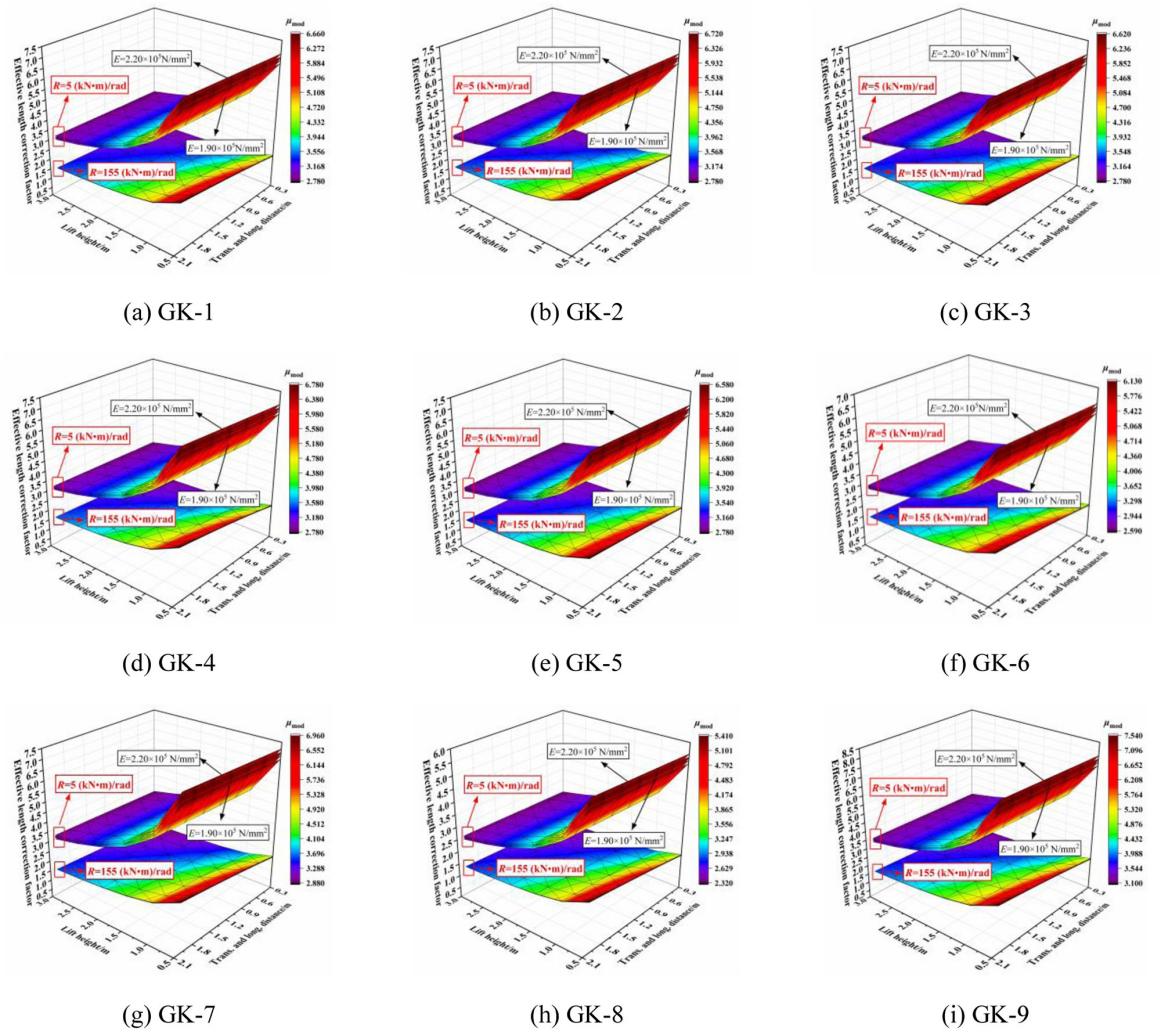
Three-dimensional diagrams of the influence of elastic modulus on the effective length correction factor. (a) GK-1. (b) GK-2. (c) GK-3. (d) GK-4. (e) GK-5. (f) GK-6. (g) GK-7. (h) GK-8. (i) GK-9.

The three-dimensional relationship between the elastic modulus and effective length correction factor is shown in Fig 27. When the joint bending stiffness was large, the elastic modulus had no influence on the effective length correction factor. With decreasing joint bending stiffness, the elastic modulus had an influence on the effective length correction factor such that the effective length correction factor was proportional to the elastic modulus. This rule was not affected by the section size of the horizontal bar and vertical pole, and the setting parameters.

## 8 Conclusions

1. When the overall high support system bears the initial load, the parameters of lift height, both big or small, have a significant influence on the effective length correction factor. This influence was more obvious when the transverse and longitudinal distance was large. When the load of the overall high support system decreases gradually, the parameters of lift height play a decisive role in the effective length correction factor, and the parameters of transverse and longitudinal distance have little influence.
2. When the joint bending stiffness is less than 100 (kN·m)/rad, the effective length correction factor is greatly affected by the joint bending stiffness. With increasing of joint bending stiffness, the effective length correction factor decreases rapidly and the transverse and longitudinal distance has no influence. When the joint bending stiffness is increased to greater than 100 (kN·m)/rad, the effective length correction factor remains unchanged and the transverse and longitudinal distance has a negligible influence on the effective length correction factor. The bending stiffness of the relative dangerous condition is set to *R* = 150 (kN·m)/rad, and the bending stiffness of the relative dangerous condition is set to *R* = 100 (kN·m)/rad. Finally, the effective length correction factor of different working conditions are obtained.
3. When the joint bending stiffness is large, the effective length correction factor is affected primarily by the section size of the outer diameter. This influence of the wall thickness is small. The effective length correction factor decreases with increasing outer diameter size of horizontal bar. When the joint bending stiffness is small, the effective length correction factor is affected primarily by the section size of vertical pole, especially the outer diameter size. The effective length correction factor increases with increasing section size of the vertical pole. Therefore, it is known that the outer diameter size of the horizontal bar plays a decisive role in the effective length correction factor.
4. When the joint bending stiffness is large, at the initial stage of the overall structure under loading, the outer diameter size of the horizontal bar and the vertical pole section influence the effective length and the wall thickness size of the section has little influence. A larger outer diameter size of the horizontal bar section is more conducive to the overall stability. When the joint bending stiffness is small, i.e., the overall structure is in a failure state, the section size of the vertical pole has an influence on the effective length and the section size of the horizontal bar has little influence. The effective length increases with increasing section size of the vertical pole.
5. When the joint bending stiffness is large, the elastic modulus has no influence on the effective length correction factor. With decreasing joint bending stiffness, the elastic modulus has a proportional influence on the effective length correction factor.

## References

[1] Ministry of Housing and Urban and Rural Construction of the People’s Republic of China. JGJ231-2010. Technical specification for safety of disk lock steel tubular scaffold in construction. Beijing: China Building Industry Press, 2010.

[2] Ministry of Housing and Urban and Rural Construction of the People’s Republic of China. JGJ231-2021. Technical standard for safety of disk lock steel tubular scaffold in construction. Beijing: China Building Industry Press, 2021.

[3] Luo YZ, Zheng YF, Xie JQ, Chen H, Shi BH. and Xiao XW. Classification and stability analysis on temporary support structures in construction. Journal of Building Structures.2016 Apr 05;37(04):143–150.

[4] Zhang H, Su MZ, Cheng QQ. and Li TF. Stability capacity of single-story non-sway frame with uniform beam loading. Progress in Steel Building Structures. 2019 Apr;21(04):94–102.

[5] Wang KF. and Li H. Research on the stability bearing capacity of semi-rigid steel frame columns. Steel Construction. 2018 Feb 22;33(02):19–25.

[6] Xie XY, Chen G. and Yin L. Multi-parameter simulation method of semi-rigid node of steel tubular scaffold with couplers. Journal of Civil and Environmental Engineering. 2019 Jun 14;41(04):92–103.

[7] Zhao ZW, Liu HQ, Dong JF, and Bian YX. Buckling capacity of socket-template scaffold system without X-bracing. Journal of Performance of Constructed Facilities, 2020 Feb;34(01), 04019089:1–14.

[8] Chen GX, Guo ZQ, Hu DP. and Xu C. Study on applicability of torque-rotation model of disk-pin joint node of disk lock steel tubular scaffold. J. Xi’an Univ. of Arch. & Tech. (Natural Science Edition). 2020 Apr 28;52(02):192–199+232.

[9] Yong C, Yong G. and Xu HW. Effective length factor of a non-symmetrical cross-bracing system with a discontinuous diagonal. Journal of Zhejiang University-Science A (Applied Physics & Engineering).2019;20(08):590–600.

[10] Rezaiee-Pajand Mohammad, Shahabian Farzad, Bambaeechee Mohsen. Buckling analysis of semi-rigid gabled frames. Structural Engineering and Mechanics. 2015;55(03):605–638.

[11] Na LY, Zhao Y, Su YQ. Stability analysis of single tube column and lattice column combined support frame system. Progress in Steel Building Structures. 2020 Feb;22(02):101–110.

[12] Zeng FK, Hu CM, Ge ZS. and Yan X. Study on value of effective length coefficient of upright stanchion of coupler steel tube falsework. Industrial Construction. 2010 Feb 20;40(02):24–27.

[13] He XP, Zhu K, Chen Y, Chang DM. and Liang Y. Analysis and research of testing about right-angle fastener of steel pipe support on the mechanics properties under torsional force and shear force. J. Xi’an Univ. of Arch. & Tech. (Natural Science Edition). 2016 Feb 28;48(01):47–51+57.

[14] Chen ZH, Lu ZR, Wang XD, Liu HB. and Liu Q. Experimental and theoretical research on capacity of unbraced steel tubular formwork support based on sway frame with semi-rigid connection theory [J]. Journal of Building Structures. 2010 Dec 05;31(12):56–63.

[15] Chen ZH, Lu ZR. and Wang XD. Numerical analysis and experimental study of the stiffness of right angle couplers in tubular steel scaffolds. China Civil Engineering Journal, 2010 Sep 15;43(09):100–108.

[16] Lu ZR, Chen ZH, Wang XD, Liu Q. and Liu HB. Experimental and theoretical study of the bearing capacity of fastener steel tube full-hall formwork support system [J]. China Civil Engineering Journal, 2012 Jan 15;45(01):49–60.

[17] Lu ZR, Chen ZH, Wang XD, Guo C. and Liu Q. Study of the bearing capacity of fastener steel tube full hall formwork support using the theory of stability of pressed pole with three-point rotation restraint. China Civil Engineering Journal. 2012 May 15;45(05):104–113.

[18] Slimani A., Ammari F., Adman R. The effective length factor of columns in unsymmetrical frames asymmetrically loaded. Asian Journal of Civil Engineering. 2018 Mar 31;19(04):487–499.

[19] Tian WF, Sun JL, Hao JP. Effective length factors of three- and two-segment stepped columns. Journal of Constructional Steel Research. 2021 Jun;181, 106585.

[20] Tian WF, Sun JL, Hao JP. Effective length factors of stepped columns considering interaction effect among columns. Journal of Building Structures, 2021 Sep;42(09):159–171.

[21] Shuang C, Zhou DH, Lan SW, and Chen J. Simple calculation of critical bearing capacity of sway frame. J. Huazhong Univ. of Sci. & Tech. (Natural Science Edition). 2019 Aug 09;47(08):55–59.

[22] Lan SW, Zhou DH., Shuang C. Calculation method of critical force of sway frame based on deflection method. J. Huazhong Univ. of Sci. & Tech. (Natural Science Edition).2019 May 15;47(05):122–127.

[23] Dong JF, Liu HQ, Xia SY, Cheng Y, Lei M and Chen ZM. Experimental research and finite element analysis on structural stability of disc-buckle type formwork support. International Journal of Steel Structures. 2022 Jun; 22(03), 748–766.

[24] Dong JF, Liu HQ, and Zhao ZW. Buckling behavior of a wheel coupler high-formwork support system based on semi-rigid connection joints. Advanced Steel Construction, 2022 Mar, 18(01), 425–435.

[25] Zhao ZW, Liu HQ, Liang B, and Yan RZ. Influence of random geometrical imperfection on stability of single-layer reticulated domes with semi-rigid connection. Advanced Steel Construction. 2019 Mar;15(01):93–99.

[26] Zhao ZW, Liu HQ, Liang B. Novel numerical method for the analysis of semi-rigid jointed lattice shell structures considering plasticity. Advances in Engineering Software. 2017 Dec;114:208–214.

[27] Zhao ZW, Zhang HW, Xian LN, and Liu HQ. Tensile strength of Q345 steel with random pitting corrosion based on numerical analysis. Thin-walled structures. 2020 Mar;148, 106579.

[28] Qian XJ. Experimental research and technical application of joint mechanical behavior of disk lock and cuplok steel tubular falsework. Nanjing: Southeast University. 2016.

[29] Du RJ. Scientizing and standarding regulations on design and calculation of construction falsework in scaffold structure (2). Construction Technology. 2010 Feb 18; 39(02), 110–116.

